# The potential of food environment policies to reduce socioeconomic inequalities in diets and to improve healthy diets among lower socioeconomic groups: an umbrella review

**DOI:** 10.1186/s12889-022-12827-4

**Published:** 2022-03-04

**Authors:** Anne Lene Løvhaug, Sabrina Ionata Granheim, Sanne K. Djojosoeparto, Janas M. Harrington, Carlijn B. M. Kamphuis, Maartje P. Poelman, Gun Roos, Alexia Sawyer, Karien Stronks, Liv Elin Torheim, Cliona Twohig, Stefanie Vandevijvere, Frank J. van Lenthe, Laura Terragni

**Affiliations:** 1 Department of Nursing and Health Promotion, OsloMet – Oslo Metropolitan University, Oslo, Norway; 2 Department of Public Health and Sport Sciences, Inland Norway University of Applied Sciences, Hamar, Norway; 3 Department of Human Geography and Spatial Planning, Faculty of Geosciences, Utrecht University, Utrecht, Netherlands; 4 HRB Centre for Health and Diet Research, School of Public Health, University College Cork, Cork, Ireland; 5 Department of Interdisciplinary Social Science, Utrecht University, Utrecht, Netherlands; 6 Chair group Consumption and Healthy Lifestyles, Wageningen University and Research, Wageningen, Netherlands; 7 Consumption Research Norway (SIFO), OsloMet – Oslo Metropolitan University, Oslo, Norway; 8 Department of Public and Occupational Health, Amsterdam University Medical Centres, University of Amsterdam, Amsterdam, Netherlands; 9 Sciensano, Departement of epidemiology and public health, Brussel, Belgium; 10 Department of Public Health, Erasmus University Medical Center Rotterdam, Rotterdam, Netherlands

**Keywords:** Nutrition policies, Food environments, Diets, Socioeconomic inequalities, Umbrella review

## Abstract

Socioeconomic inequalities in diets need to be tackled to improve population diets and prevent obesity and diet-related non-communicable diseases. The potential of food environment policies to reduce such inequalities has to date however not been appraised. The objective of this umbrella review was to assess the impact of food environment policies on socioeconomic inequalities in diets and to identify knowledge gaps in the existing literature, using the Healthy Food Environment Policy Index as a conceptual framework. The policies considered in the umbrella review are within six domains: 1) food composition 2) food labelling 3) food promotion 4) food provision 5) food retail 6) food pricing. A systematic search for systematic literature reviews on the effect of food environment policies on dietary-related outcomes across socioeconomic groups and published in English between 2004 and 2019 was conducted. Sixteen systematic literature reviews encompassing 159 primary studies were included, covering food composition (*n* = 2), food labelling (*n* = 3), food provision (*n* = 2), food prices (*n* = 13) and food in retail (*n* = 4). Quality assessment using the “Assessing the Methodological Quality of Systematic Reviews” quality rating scale showed that review quality was mainly low or critically low. Results suggest that food taxation may reduce socioeconomic inequalities in diets. For all other policy areas, the evidence base was poor. Current research largely fails to provide good quality evidence on impacts of food environment policies on socioeconomic inequalities in diets. Research to fill this knowledge gap is urgently needed.

**Supplementary Information:**

The online version contains supplementary material available at 10.1186/s12889-022-12827-4.

## Background

Overweight and obesity have rapidly become a major public health challenge [1]. Unhealthy diets are a main risk factor for overweight and obesity and for diet-related non-communicable diseases (NCDs) such as type 2 diabetes, several types of cancer and cardiovascular diseases [2]. People with a lower socioeconomic position (SEP), measured according to e.g. income, education or occupation, are less likely to consume diets in line with dietary recommendations and are more likely to become overweight or obese [3]. Drawing on definitions of health inequalities [4], we use the term socioeconomic inequalities in diets to indicate systematic differences in dietary quality between different population groups, linked to their socioeconomic position in society.

Food environments are an important determinant for people’s diets and may influence socioeconomic inequalities in diets [3, 5]. Food environments have been defined as the collective physical, economic, policy and sociocultural surroundings, opportunities and conditions that influence people’s food and beverage choices and nutritional status [6]. Several studies have indicated that SEP mediates how dietary behaviors are influenced by the food environment: lack of economic resources may reduce the affordability of healthy food, and low literacy may hinder comprehension of health information and food labels [3, 5]. Moreover, groups with lower SEP have been found to be relatively more exposed to unhealthy food environments [5, 7, 8]. In order to reduce socioeconomic inequalities in diets and achieve global, regional and national nutrition and NCD goals [9–11] it is imperative to promote healthy food environments focusing particularly on lower SEP groups.

Governments have the most impactful role in promoting healthy food environments, through a range of policy measures in areas such as food labelling and taxation, among others [12]. The International Network for Food and Obesity/NCDs Research, Monitoring and Action Support (INFORMAS) has gained acknowledgment for developing a framework to assess and monitor different dimensions of food environments, aiming to increase their healthiness and prevent obesity, diet related NCDs and related socioeconomic inequalities [6]. Within this framework, the Healthy Food Environment Policy Index (Food-EPI) was developed to monitor the implementation of food environment policies compared to international best practices at national, supranational or local levels [13] and this framework was used to guide our study. The Food-EPI includes seven policy domains that facilitate the accessibility, availability and affordability of healthy foods: 1) food composition, 2) food labelling, 3) food promotion, 4) food provision, 5) food retail, 6) food prices, and 7) food trade and investment [13]. Table 1 provides descriptions of policies related to each domain.

**Table 1:** Food-EPI policy domains

Policy domain	Description
**Food composition**	Policies or standards to improve the nutritional quality of the food supply, in particular processed foods and out-of-home meals, e.g., maximum sodium levels, trans fat ban, sugar reduction schemes.
**Food labelling**	Policies on food labelling to help consumers make healthier, informed choices, e.g., standards for ingredient lists/nutrient declarations; health and nutrition claims; front-of-pack labelling schemes and menu labelling.
**Food promotion**	Policies that restrict unhealthy food promotion (marketing) to children and adolescents across relevant media and contexts (i.e., broadcast media, online and social media, non-broadcast media, settings where children gather and on food packages).
**Food prices**	Economic tools to incentivize healthy food purchases and disincentive unhealthy food purchases (food taxes and subsidies); food-related income support programs aimed at low SEP groups.
**Food provision**	Policies to promote healthy foods in schools and other public settings, e.g. nutrition standards for school meals; government-developed guidelines and support systems for food provision (for employees) in private companies
**Food retail**	Policies to improve access to healthy food and limit access to unhealthy foods in communities (e.g., zoning laws). Government-developed guidelines and support systems, targeted at the private sector, to promote healthier foods within food outlets or restaurants.
**Food trade and investment**^**a**^	Measures to identify and minimize negative impacts of trade agreements on public health and nutrition and protect governmental regulatory capacity in relation to investments that may impact public health.

Descriptions of food environment policy domains included in the Healthy Food Environment Policy Index (Food-EPI) (adapted from Swinburn 2013). ^a^ The Food trade and investment policy domain has not been considered in this study

Policies in these domains may have different impacts on the diets of lower and higher SEP groups. To support government decision-making, it is important to evaluate food environment policies in terms of their impact on socioeconomic inequalities in diets [14]. In addition, it is important to assess the potential of programs and interventions that can inform further policy development. A number of systematic reviews on food and nutrition policies that include an inequality perspective have been published the last decade, in particular regarding food pricing policies [15–17]. In a recent umbrella review, Thomson et al. reviewed a broad range of public health policies on health inequalities in high-income countries [18]. They assessed both primary and secondary prevention policies according to different delivery mechanisms (e.g., fiscal, regulation and education within primary prevention) and across eight policy domains including food and nutrition policies [18]. According to this study, taxes for unhealthy foods and targeted food subsidy programs, both fiscal delivery mechanisms, were effective in reducing health inequalities. On the other hand, free fruit provision in schools, another fiscal mechanism, did not influence health inequalities. For regulatory mechanisms, policies for salt reduction and trans-fat restrictions were found to be equally effective in all socioeconomic groups and therefore did not influence inequalities in outcomes. For calorie labelling on menus, mixed results were reported [18].

However, to the best of our knowledge, no systematic summary of the evidence that specifically focus on the effects of food environment policies on socioeconomic inequalities in diets has been produced to date.

The aim of this study is therefore to conduct an umbrella review on the evidence of the impact of food environment policies and interventions on socioeconomic inequalities in diets, according to the following Food- EPI policy domains: food composition, food labelling, food promotion, food prices, food provision and food retail (Table 1). Aligning with existing recommendations [19] we wanted to both assess differential impacts of policies and interventions across levels of SEP and assess impacts on low SEP groups specifically. In addition, we also aimed to identify knowledge gaps in the evidence.

## Methods

Umbrella reviews integrate the findings of multiple systematic reviews and allow rapid review of the evidence base in relation to a topic [20, 21]. Reporting guidelines for umbrella reviews are currently under development [22, 23]. Therefore, this study follows the Preferred Reporting Items for Systematic Reviews and Meta-Analyses (PRISMA) Statement [24]. A PRISMA checklist is available in Additional file 1. A review protocol for this umbrella review was registered in the International prospective register of systematic reviews (PROSPERO) [25], registration number CRD42020154855.

### Eligibility

Reviews had to meet the following inclusion criteria:

- **Population and setting:** Groups from the general population (children [up to 18 years], adults and elderly) from any geographical region or country income group (high, middle and low-income countries).
- **Intervention/exposure:** Experimental and non-experimental studies that evaluated policies or research interventions according to the Food-EPI domains. For the purpose of simplicity and readability we will use the term *policy interventions*, to include policies, research-initiated interventions, actions or programs that change one or more domains of the food environment. Policy interventions could be implemented or tested at the national, local, community or organizational level.
- **Comparison:** In line with other reviews [26], comparison was interpreted in the context of SEP. Eligible reviews reported:

(i) outcomes across different SEP groups; *or*
(ii) outcomes specifically for lower SEP groups; *or*
(iii) both.

Accepted SEP measures were based on recommendations from health equity literature [19, 27] and other reviews [18, 26] and included education, income, occupation and area deprivation. To avoid missing relevant studies that did not use rigourous reporting standards (i.e. PRISMA-E or the PROGRESS framework [19, 27], we accepted reviews which did not focus solely on inequalities, as long as sufficient data on inequality aspects could be extracted from the results section.

- **Outcome:** these were related to dietary behavior and included dietary intake (measured at the nutrient level, amount or frequency of food consumption), food purchase, expenditure and spending, as well as price elasticity (a measure of the change in the quantity demanded or purchased of a product in relation to its price change).
- **Study design:** Systematic literature reviews, umbrella reviews and scoping reviews were eligible.
- **Time range:** Reviews published in the past 15 years (2004–2019). We anticipated that relevant reviews had mainly been published the last 10 years (following e.g., the recommendations of the World Conference on Social determinants of Health in 2011 [14] and added five extra years to ensure earlier relevant reviews were captured.
- **Language:** English.

We excluded reviews that assessed the effect on inpatient (hospitalized) groups and reviews that assessed outcomes such as knowledge, awareness, or intention to buy or nutritional status indicators such as micronutrient deficiencies.

### Search strategy

We defined the search terms with the guidance from a university search librarian and informed by related systematic reviews [15–17], combining free text and subject headings search terms for four categories: (i) relevant policy interventions (ii) food and diet; (iii) socioeconomic inequality; (iv) study type. A separate search strategy was developed for the food promotion policy domain because specific search terms on children and adolescents were necessary. The search strategies were piloted and tailored to the different databases. An example search string from Medline is available in Additional file 2. Searches were undertaken in Medline (Ovid), Embase (Ovid), Web of Science, Food Science Source (EBSCOhost), Epistemonikos and Cochrane Library in September 2019. An additional search was undertaken in November 2019 in the interdisciplinary database Scopus to ensure coverage of journals within the social sciences. An email alert was set up in Web of Science to identify reviews that were published after the search had been conducted, and alerts were assessed in February 2020.

### Study selection and data extraction

Four reviewers were involved in the screening process (ALL, SIG, LT, GR). Each record was screened by two independent reviewers. At the initial screening of titles and abstracts, ALL reviewed the full list of records while SIG, LT and GR screened 1/3 each of the list, using the Rayyan QCRI software [28] to manage the screening process. Discrepancies at this stage were resolved by a third reviewer within the team. At the full text screening stage, all four reviewers screened the ten first records to refine the inclusion criteria, in particular regarding inclusion based on SEP. The records were then divided between the four reviewers (1/2 of the record list to each), ensuring all full text records were screened independently by two reviewers. They used an agreed guidance for full-text screening to ensure consistency in screening (available in Additional file 3). Discrepancies at this stage were resolved by consensus or by involving an additional reviewer (LET).

Data extraction forms were pre-designed and piloted as a part of this study, including items such as: search time frame of study, study design(s), population, framing of inequality, policy interventions, number of relevant studies in review; summary of results, and (if possible) notes on the direction of the effect on socioeconomic inequalities in diets, further described below. A sample of the data extraction form is available in Additional file 4. The model for the data extraction process was developed based on earlier umbrella and systematic reviews [26, 29, 30]. ALL extracted data from all included reviews and another reviewer checked the data (JH, CT, SKD or MP). Results were extracted based on the highest possible level of detail in each review, e.g. by extracting reported results per primary paper and not only overall conclusions. If additional detail was needed to extract data, supplementary material (but not primary studies) was consulted. This was done for four reviews [16, 31–33]. Any disagreements were resolved by consensus.

### Quality assessment

Quality assessment was performed using the Assessing the Methodological Quality of Systematic Reviews quality assessment checklist (AMSTAR 2), which is developed for systematic reviews of both randomized and/or non-randomized studies [34]. In order to facilitate and simplify assessment of a range of study designs we made minor revisions to AMSTAR 2 items (available in Additional file 5). The quality assessment was performed in duplicate by AS, CBMK and KS and discrepancies solved by CT. As recommended by AMSTAR 2, a set of critical items from the checklist were agreed on and used as a basis for rating the overall confidence in the results of each review (Additional file 6). These items included e.g., “satisfactory assessment of risk of bias in individual studies” (item 9) and “risk of bias accounted for in interpretation of results in the review” (item 13). Based on the number of critical and non-critical flaws, included reviews were categorized as having high, moderate, low or critically low quality.

### Analysis

The reviews were narratively synthesized, using the Food-EPI policy domains as a conceptual framework for presentation of results. In terms of differential effect on SEP, whenever possible we used the strategy employed by Olstad et al. [17] to describe the overall direction of results for each policy intervention:

(i) **Positive**: the effect of policy intervention was larger in lower vs. higher SEP groups, thus contributing to reduce socioeconomic inequalities in diets.
(ii) **Neutral:** there was no difference in effects in outcomes across SEP.
(iii) **Negative:** the effect of the policy intervention was higher in higher vs. lower SEP groups, thus contributing to increase socioeconomic inequalities in diets.
(iv) **Inconclusive or no effect:** the results were inconsistent, or no effect of policy intervention was detected.

For the results that were specific for low SEP groups, the direction of results was interpreted in terms of the absolute effect of the policy intervention on the outcomes and described as positive (healthier dietary outcomes), negative (unhealthier dietary outcomes) or having no effect.

## Results

**Fig. 1:**
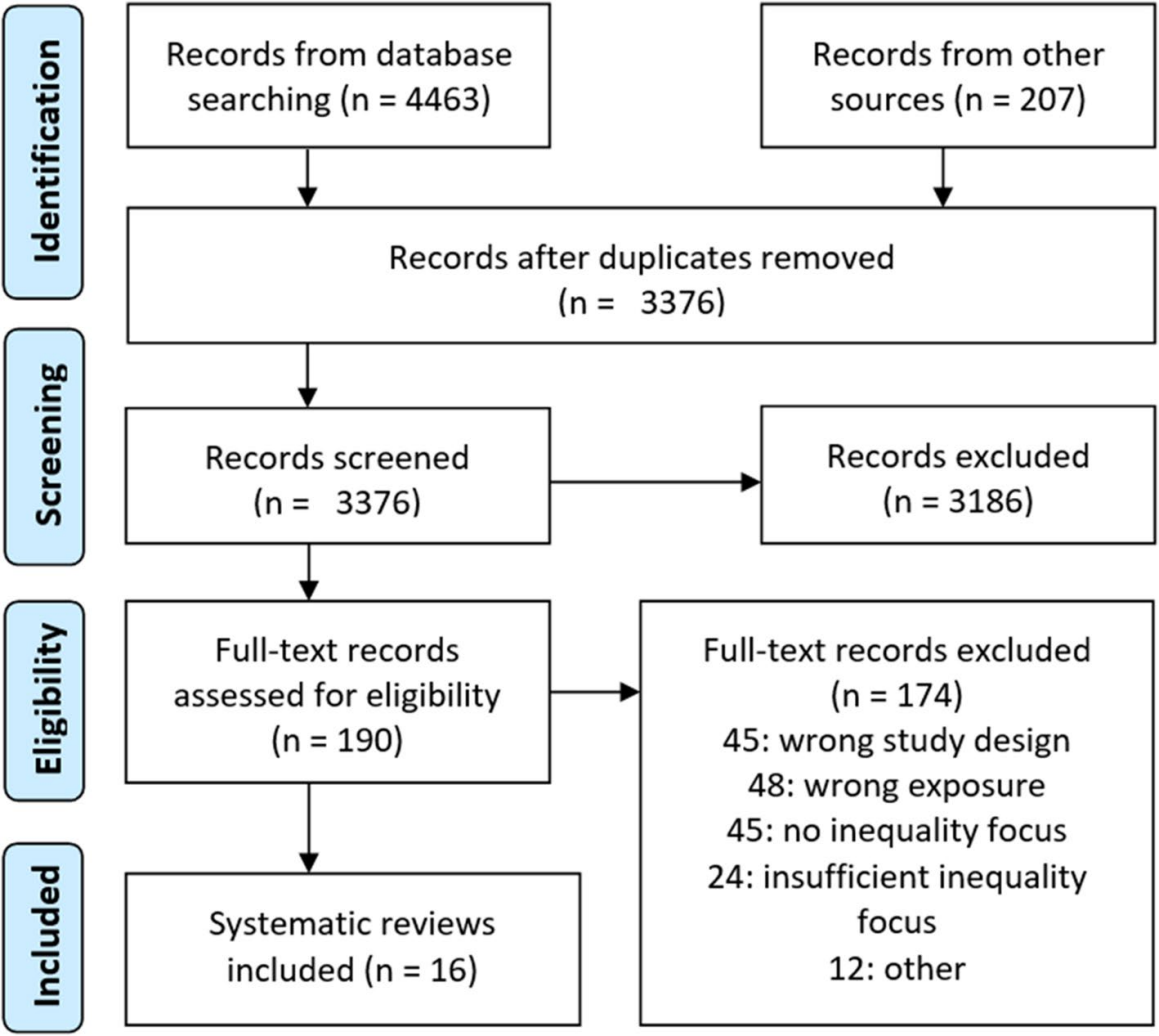
PRISMA flowchart. Preferred Reporting Items for Systematic Reviews and Meta-Analyses (PRISMA) flowchart. From: Moher D, Liberati A, Tetzlaff J, Altman DG, The PG. Preferred Reporting Items for Systematic Reviews and Meta-Analyses: The PRISMA Statement. PLoS Med. 2009;6 [7]: e1000097. 10.1371/journal.pmed.1000097

4680 records were retrieved via searches in the seven databases, search alerts and reference list screening. Those were imported into EndNote where duplicates (*n* = 1294) were removed. We screened 3376 records at the title and abstract stage and 190 records at the full text stage and included in total 16 systematic literature reviews (henceforth referred to as reviews). No relevant umbrella reviews or scoping reviews were detected. Exclusion reasons for the 174 records excluded at the full text stage are available in the Prisma flow diagram (Fig. 1) and in Additional file 7.

The included reviews were published between 2010 and 2018, and were mainly conducted in Western, high-income countries (i.e., the United States and Europe). One review focused on middle-income countries (MICs) [32] and no reviews focused on low-income countries. Thirteen reviews had an overall focus on socioeconomic inequalities, addressing inequality in the overall theme or as a main objective of the study. In three reviews [33, 35, 36] inequality issues emerged in the results section and less details could be extracted. Generally, populations were broadly described (e.g., “adults”, “households”) except two reviews [17, 37] that reported results specifically for children. In descending order, the main measures for SEP as reported in the reviews were income, area level income or deprivation, education, occupation and eligibility for targeted programs for low-income groups. Six reviews used SEP (or social class) as a composite measure without operationalizing measures or proxies. Five reviews focused explicitly on low SEP populations [37–41], three reviews reported outcomes for both low SEP groups and across SEP [16, 31, 42], and the remaining reviews reported results across SEP. Study characteristics and key findings are provided in Additional file 8. In descending order, the main outcomes covered by the reviews were dietary intake/food consumption, food purchase, and consumer demand/price elasticity. Table 2 shows how included reviews were distributed across Food-EPI policy domains. No review covering the food promotion domain was identified. The reviews included 159 unique primary studies (a full list of relevant primary studies as reported in each review is provided in Additional file 9).

**Table 2:** Review quality and policy domains assessed in the included reviews

Review	Quality^**a**^	Food compo-sition	Food labelling	Food promotion	Food prices	Food provision	Food retail
Abeykoon 2017	L						✓
Andreyeva 2010	CL				✓		
Backholer 2016	CL				✓		
Black 2012	L				✓		
Cuffey 2015	CL				✓		
Eyles 2012	L				✓		
Hartmann-Boyce 2018	L		✓		✓		✓
Hendry 2015	M	✓					
McGill 2015	L	✓			✓		
Nakhimovsky 2016	H				✓		
Olstad 2016	M		✓		✓	✓	
Olstad 2017	M				✓	✓	✓
Sarink 2016	L		✓				
Schultz 2015	CL				✓		
Thow 2010	CL				✓		
Thow 2014	L				✓		

Policy domains according to the Food-EPI framework that were covered by included reviews in the umbrella review. ^a^ Quality according to AMSTAR 2. *H* high, *M* moderate, *L* low, *C* critically low

### Quality of included reviews

Using the AMSTAR 2 tool, one review was assessed as being of high quality [32], three reviews were of moderate quality [17, 36, 37], seven reviews had low quality [16, 26, 31, 35, 38, 39, 42] and five reviews had critically low quality [15, 33, 40, 41, 43] (Table 2). Additional file 6 shows the full quality appraisal including how the reviews scored on critical items. Only two reviews were considered to have performed a fully satisfactory assessment of the risk of bias in primary studies (item 9), with ten studies receiving a “partial yes” on this item, but most studies were considered to have accounted for risk of bias in the discussion of results (item 13). The quality of the included *primary studies* was reported in the respective reviews as low to medium mainly due to study design (e.g., there were quantitatively more modelling and observational study designs than experimental designs). The quality appraisal undertaken in reviews included use of defined tools (i.e. the Effective Public Health Practice Project Quality Assessment Instrument [EPHPP] [26, 38, 42]; the Effective Practice of Care [EPOC] guidelines and the Newcastle-Ottawa Scale [39]; the Methods for Evaluating Research Guideline Evidence [MERGE] [42]; the Community Guide of the US Task Force on Community Preventive Services [26]; and several defined Cochrane checklists [17, 31, 35–37, 39]; quality checklists derived from other studies [15, 32]; quality assessment according to criteria listed by review authors [16, 32, 40, 41], and combinations of the above. One review did not report quality assessment [43].

Below, the findings of the included reviews are presented as a narrative synthesis organized according to i) Food-EPI policy domains, and ii) whether results were expressed across SEP groups or specifically for lower SEP groups. Table 3 presents the size of the evidence base (number of reviews and primary studies), overall quality of reviews, and the overall direction of results in terms of the effect on socioeconomic inequalities in diets, according to each policy domain.

**Table 3:** Overall findings

Policy domain	SEP perspective	Evidence base N^o^ systematic literature reviews (N^o^ unique studies)	Overall quality assessment of included reviews	Direction of results
**Food composition**	Across SEP	2 (2)	Low/moderate	↔
	Low SEP	0 (0)		
**Food labelling**	Across SEP	3 (7)	Low	~ / 0
	Low SEP	1 (7)	Low	0
**Food prices** *a. Food taxes*	Across SEP	8 (38)	Low	↑ ↔
	Low SEP groups	1 (12)	Low	↑
*b. Food subsidies*	Across SEP	4 (7)^a^	Low	~
	Low SEP	1 (4)	Low	↑
*c. Targeted policies*	Low SEP	4 (69)	Low	↑
**Food provision**	Across SEP	1 (9)	Moderate	↔
	Low SEP	1 (6)	Moderate	↑
**Food retail**	Across SEP	0 (0)		
	Low SEP	3 (13)	Low	~/ 0

Note: Summary of evidence base, overall quality assessment and the overall direction of results on socioeconomic inequalities in diets per policy domain. Overall quality assessment is based on the average score of systematic literature reviews within each policy domain. Promotion is absent from table as no studies were identified in this domain

↑: Positive effect (larger impact in low vs. high SEP groups)

↔: neutral effect (no difference in impact across SEP)

↓: negative effect (larger impact in high vs low-SEP groups) – not detected

~ Inconclusive results

0: No effect

When arrows are used to describe the effects for low SEP groups specifically ↓ denotes a negative, absolute effect and ↑ denotes a positive absolute effect

^a^Subsidies and combinations of taxes and subsidies

### Impact of food environment policies on socioeconomic inequalities in diets

### Food composition

This policy domain includes measures to improve the nutrient content of foods, often relating to reducing nutrients of concern such as trans- or saturated fatty acids, sodium/salt or sugar in industrially produced foods. We identified two reviews within this domain.

#### Across SEP groups

There is little evidence on the effects of food composition policy interventions across SEP groups, as the two reviews only encompassed one primary study each. McGill et al. [26] included one observational study assessing the effects of a salt reformulation policy on salt intake in the UK. Hendry et al. [36] included one observational study conducted in New York City, assessing the effects of a trans-fat ban on the mean content of trans-fat acids in customers’ lunchtime fast food purchases. Both studies showed neutral effects on socioeconomic inequalities in diets.

#### Specific for low SEP groups

No results reported.

### Food labelling

This domain concerns policies that provide consumers with nutrition information to help informing food choices. Three reviews encompassing 11 unique primary studies were identified. They evaluated two types of food labelling: front of pack (FOP) labelling and menu energy labelling.

#### Across SEP groups

The only evidence on *FOP labelling* stems from a review by Hartmann-Boyce et al. [31] which encompassed two relevant primary studies. Both were randomized controlled trials with purchase as outcome. Results were inconclusive, with one study showing negative and one study showing neutral and partly conflicting results on socioeconomic inequalities in food purchases. *Menu energy labelling* policies and their effects on food purchases were investigated by Olstad et al. [17] and Sarink et al. [42], encompassing in total five unique primary studies. All were natural experiments of policy interventions implemented in cities in the United States, with SEP measured at the area level. Three of these primary studies reported no effect of the policy intervention and two studies showed either negative or neutral effects on food purchases according to SEP. The size of effects was generally very small.

#### Specific for low SEP groups

The review by Sarink et al. [42] also presented results for low-income groups separately. Seven included primary studies generally found that menu energy labelling did not alter the energy content of food purchases made in low-income areas after policy intervention.

### Food prices

This domain concerns economic measures to incentivize healthy or disincentive unhealthy food purchases. 13 reviews within the food price domain, encompassing 120 unique primary studies, were included. We organize the results according to the three Food-EPI policy subdomains: (i) food taxes, (ii) food subsidies and (iii) food related income support programs that are specific for low SEP groups, (henceforth termed “targeted policies”).

### Food taxes

#### Across SEP groups

Eight reviews evaluated the effect of food and/or beverage taxes on a range of outcomes across SEP. In total, they encompassed 38 unique primary studies, thirteen of which were included by several reviews. Below we have strived to avoid duplication of results as far as possible and overlap between these studies can be examined in Additional files 8 and 9. The majority of included studies in the reviews were modelling studies. Overall, results suggest that food taxation would be neutral or positive in terms of their effects on socioeconomic inequalities in diets, with very few reviews reporting negative results. For example, a review by Backholer et al. [15] encompassed eight relevant primary studies (including six modelling studies) which evaluated the effect of sugar-sweetened beverage (SSB) taxes on beverage purchases, dietary intake or own price elasticity (OPE). One cross-sectional primary study showed an association between SSB tax and SSB consumption in children of low SEP but no association in the general population. One study with OPE as the main outcome reported that low-SEP vs. high SEP households were “generally” more responsive to increases in SSB prices, but the difference between the highest and lowest quintiles were not statistically significant. The six included modelling studies reported varied OPE estimates, with only one study reporting low-SEP households to be more price elastic (i.e., more responsive to a SSB tax). However, when OPEs were used as a basis to model the effects of hypothetical taxes on energy intake or SSB purchase, 5 of 6 studies showed neutral effects and one showed positive effects on socioeconomic inequalities, due to the higher baseline SSB consumption in low SEP groups. A review by Eyles et al. [16] comprised 11 modelling studies that assessed the effect of food taxes on food purchases across SEP. In this review, 5 of 11 studies showed neutral effects, 4 of 11 studies showed positive effects, one study showed negative effects, and one study had inconclusive results in terms of inequalities. Thow et al. [35] reported that 9 of 10 included modelling studies showed that low-SEP groups were more price sensitive and therefore more likely to change purchase patterns in response to a tax. Four of the primary studies in this review had also been reported in the reviews by Eyles et al. or Backholer et al. Olstad et al. [17] included two relevant cross-sectional studies, one of which was not reported by other reviews. This study assessed an implemented tax on unhealthy foods in Hungary and showed a positive impact on socioeconomic inequalities in food purchases. McGill et al. [26] included four relevant studies where one was not reported by other reviews. This study was a randomized controlled trial (RCT) assessing the effect of a high (50%) tax on high energy density food which showed a positive effect on inequality in calories purchased.

Nakhimovsky [32] included the only evaluation of taxation impacts on inequality in *Middle-income countries* (Mexico [*n* = 3], India, Brazil and Ecuador). Overall, this review indicated that low SEP groups were relatively more responsive to price changes in SSB products compared to higher SEP groups. Four of 6 studies reported positive results for socioeconomic inequalities in food purchases or reported that own price elasticity was higher in low vs. high SEP groups, whereas 2 of 6 studies, one of which a modelling study, reported neutral results.

Two reviews, both from 2010, were less informative. One did not manage to identify consistent differences in estimated own price elasticities between SEP groups due to the low numbers of relevant studies identified [43]. Another review reported that the effects of food taxes were *economically* regressive (meaning that low SEP groups will pay more in food taxes compared to high SEP groups) [33].

#### Specific for low SEP groups

Eyles et al. [16] was the only review reporting absolute results for the effects of food taxation policies for low-SEP populations specifically. Ten out of 12 relevant primary studies reported positive results on food purchases of taxed unhealthy items (i.e., low SEP groups reduced purchases of these items), two studies were categorized as having no impact, and one study showed varied results.

### Food subsidies

Subsidizing healthy foods such as fruit and vegetables (F&V) is one subdomain of the Food-EPI framework. We identified two reviews that evaluated the effect of universal food subsidies for healthy foods, and two reviews that reported on the effects of *taxes and subsidies in combination*. Results are inconclusive with regards to diet inequalities.

#### Across SEP groups

Two reviews including five unique primary studies assessed the effects of food subsidies for healthy foods on food purchases across SEP [31, 35]. The included studies varied in terms of study design (two were modelling studies), targeted items, and the size of subsidy varied from 3 to 30%. Thow et al. [35] concluded that universal subsidies for healthy foods (classified within “healthy food categories” or F&V) may disproportionately benefit high-income households and therefore be negative in terms of inequality. A neutral result was reported in the review by Hartmann-Boyce et al. [31] which encompassed two experimental studies on price reductions conducted in retail settings (one targeting F&V and one targeting “core foods” that met the nutrition criteria of an endorsement food labelling scheme).

McGill et al. [26] and Thow et al. [33] reported on the effects of *subsidies and taxes in combination*, such as higher prices on saturated fat or read meat combined with subsidies for fiber or F&V on dietary intake and food purchases. They encompassed in total three unique modelling studies. Only one study that combined taxation of “less healthy” foods with subsidies for F&V showed statistically significant, positive results on dietary intake.

#### Specific for low SEP groups

The review by Hartmann-Boyce et al. [31] encompassed four relevant studies that investigated the effect of subsidies for various foods defined as healthy in the form of price discounts on food purchase outcomes. These were RCTs undertaken in real (3 studies) or simulated (1 study) retail settings in study populations recruited from low SEP groups. Results from the four studies suggest that in the retail context, subsidies may have at least partial positive effects on healthy food purchases in low SEP groups.

### Targeted policies

#### Specific for low SEP groups

Four reviews investigated the effects of policies specifically targeting low SEP groups, including 69 unique primary studies. The reviews mainly included interventions implemented in the United States, such as the Supplemental Nutrition Assistance Program (SNAP) and Special Supplemental Nutrition Program for Women, Infants, and Children (WIC). The direction of results from these reviews tentatively suggest that food-related income support programs may have a small, positive impact on food purchases and diet in eligible low SEP populations.

Black et al. [39] included two primary studies assessing the effects of food subsidies within the WIC program. Both showed positive results on F&V intake in the intervention groups. Shultz et al. [41] assessed effects of revisions on the WIC scheme (targeting e.g., F&V, whole grains, and low-fat dairy) on dietary intake and food purchases. Seven primary studies found positive, albeit small changes in food intake or in food purchases in the target group after revision of eligible foods. Cuffey et al. [40] investigated the potential impact of reducing the set of SNAP eligible foods (e.g., not allowing purchase of sugar-sweetened beverages with SNAP benefits) on expenditures for restricted foods. The review included 59 primary studies and results suggested that restrictions on food items might have a small to moderate positive effect on household purchasing of restricted foods. In contrast, one single primary study in the review by Olstad et al. [37], assessing the effect of food subsidies for F&V in a farmer’s market context, did not find any effect on dietary intake.

### Food provision

This policy domain relates to measures to promote healthier foods in schools and other public settings. We identified two reviews within this category where one focused on universal policies and one focused on policies implemented in low SEP settings, both in schools. These were the only reviews considering child populations. In total they encompassed 15 unique primary studies with wide variation in interventions and the contexts in which they were implemented. In some studies, policy measures were one of several intervention components.

#### Across SEP groups

In a review from 2016, Olstad et al. [17] assessed the effect of universal nutrition policy interventions implemented in schools. Policy interventions were initiated by local or national governments and conducted in several countries, including Norway, UK and the Netherlands. Nine primary studies assessed the effect of school nutrition policies, such as free fruit schemes, meal programmes and food standards on dietary outcomes. Results showed mainly neutral results on socioeconomic inequalities, with 6 of 9 studies showing neutral results whereas the remaining three studies showed negative results in terms of inequality.

#### Specific for low SEP groups

Olstad et al. [37] investigated the effect of food provision policies implemented in socioeconomically disadvantaged populations, i.e. schools in low-income areas. This review included both evaluations of implemented policies or programmes and research-led interventions. The results suggest at least partly positive effects for diet-related outcomes in the target groups. The review included two studies on free fruit schemes that reported positive results on dietary intake (mainly assessing fruit and vegetable intake in and out of school). In addition, four studies assessed school food policies that were implemented within multicomponent interventions. Results showed positive results for at least one of the dietary intake outcomes assessed. The positive impact ranged from 10 to 50% impact, meaning for example that a 50% impact had showed positive outcomes for fruit but not for vegetable intake. For these studies it is challenging to attribute effects to one discreet intervention component.

### Food retail

This domain encompasses policies or guidelines promoting access to healthier food in communities, such as zoning laws supporting healthy food outlets/restricting unhealthy ones, or guidelines to promote healthier foods within food outlets or restaurants. We identified three reviews within this category, which included 13 unique primary studies. They all focused on low-SEP areas exclusively.

#### Across SEP groups

No results reported.

#### Specific for low SEP groups

Abeykoon et al. [38] and Olstad et al. [37], which included ten unique studies, investigated policy interventions such as opening of new grocery stores in low-income neighbourhoods with little access to healthy food outlets. Studies were conducted in England, Scotland and the United States and evaluated government- and researcher-initiated interventions on food intake and -purchase. Results were inconclusive: whereas 5 of 10 studies detected positive effects for a limited set of outcomes, with generally very small effect sizes, the remaining 5 largely failed to detect any effects.

The review by Hartmann-Boyce et al. [31] investigated environment changes inside grocery stores through alterations in item availability and/or store environment, such as signage or brochures. The review included three primary studies. One study detected significant effects on a limited set of purchase related outcomes, while the two other failed to detect significant outcomes.

## Discussion

The aim of this umbrella review was to assess and summarize evidence, at systematic review level, of food environment policies on socioeconomic inequalities in diets and to identify knowledge gaps. We included systematic literature reviews assessing the impacts of policies across levels of SEP or targeting low SEP groups specifically and identified 16 systematic literature reviews on the effects of food environment policies covering five Food-EPI domains.

Despite the inclusion of 16 relevant reviews, a major finding of our study is that the research gap in this field is substantial. As shown in Table 2 the scope of the considered policies is limited, with most included reviews considering the food pricing policy domain. The missing focus on socioeconomic inequalities in the literature at systematic review level could simply be due to a lack of implemented policies or that existing policies have not been evaluated. It is however worth mentioning that in our study we had to exclude nearly 70 reviews that evaluated relevant policy interventions as they contained no, or not sufficient, information on effects according to socioeconomic position (Additional file 7). This could be caused by systematic reviews failing to report relevant outcomes. However, as also pointed out by Thomson et al. [18], it is more likely that the gap at review level reflects an evidence base that does not adequately address inequality perspectives. This could pertain to features of study design and reporting. If primary studies lack power in their population sizes, it may hinder analysis according to SEP. It is also possible that authors do not follow reporting recommendations as provided e.g. in the PRISMA-E or PROGRESS framework [19, 27] so that relevant primary studies are not identified. More research that includes inequality perspectives in assessing food policies is urgently needed.

Food taxes was the policy subdomain with the largest evidence base (eight reviews and 38 primary studies). Results showed that taxation of unhealthy foods and beverages may have a positive effect (thus reducing inequalities) or neutral effect on socioeconomic inequalities in diets. Price elasticity were often reported to be larger in lower SEP groups, indicating that this type of policy has the potential to reduce inequalities in diet or to influence diets positively in low-SEP groups. Our findings support and strengthen the conclusions in the umbrella review by Thomson et al. [18], which suggested that taxes were effective in reducing health inequalities but was based on more limited evidence. The findings from our study are encouraging given that food and/or beverage taxation is gaining momentum internationally [44] and is also promoted as cost-effective interventions by international organizations such as the World Health Organization (WHO) and the World Bank [45, 46]. It should however be noted that the evidence was mainly based on modeling studies with high risk of bias. In addition, several primary studies were included in more than one review. Thus, the interpretation of the size of the evidence base and the strength of results should be considered with caution.

Our review also found some evidence suggesting that food-related income support programs for low SEP groups may be positive for diet-related outcomes in the target groups by increasing the affordability of healthy foods or reducing access to unhealthy foods [3]. This is in line with the umbrella review by Thomson et al. [18].

Given that school food policies are widely implemented in Europe [47], the limited number of systematic reviews with an inequality perspective that were identified on our study – only two – is remarkable. A recent overview of systematic reviews on primary obesity prevention among adolescents also noted a virtual absence of evidence on socioeconomic inequalities in the body of literature [48]. The evidence tentatively suggests that universal school food policies do not influence socioeconomic inequalities in diets (even though effects may be positive across all SEP groups), and that policies targeted at low-SEP groups may have positive implications for diet-related outcomes, as was also reported in the umbrella review by Thomson et al. [18].

Regarding food labelling, the few reviews identified were related to FOP and menu labelling only, largely showing no or inconsistent effect, in line with the findings in the umbrella review by Thomson et al. [18]. According to a WHO report, the research on FOP labeling mainly encompasses evidence on consumer use and understanding rather than food purchase or dietary intake [49] which may partly explain the limited evidence for the outcomes assessed in our study. Consumers of lower SEP are said to have poor use and understanding of the complex information found on ingredient lists and nutrition declarations [49], and simple FOP labelling schemes have been suggested as the most promising for helping all consumers understand nutritional quality [50]. At EU level, work is under way to develop a harmonized mandatory FOP nutrition labelling [51]. This process should include new investigations into the effects of FOP labels according to socioeconomic position.

The evidence available for the effects of food composition policies was very limited, with only two reviews (which included two primary studies) identified. Results from these studies were also reported by Thomson et al. [18] and suggested neutral effects on dietary inequalities, i.e., similar reductions across SEP groups. Since groups of lower SEP tend to have a relatively higher intake of food items high in saturated fat, sugar and/or salt [3], the “neutral” results may suggest contribution of these policies to reducing socioeconomic inequalities. To have a meaningful impact on dietary outcomes, policies or reformulation need to be implemented systematically and across a wide scope of foods, including those more frequently consumed in lower SEP groups [52]. A range of national policies (both mandatory and voluntary) on food composition has been implemented in both high-income and low-and middle-income countries, including standards on salt, trans-fat and to a less extent, sugar reduction [53]. However, as our results illustrate, evaluations of these policy actions are largely lacking.

Lastly, no systematic reviews were found that assessed the effects of policies to restrict food promotion of unhealthy foods to children and adolescents with a focus on socioeconomic inequalities. Promotion of unhealthy food is considered an important contributor to childhood overweigh and obesity [54]. The NOURISHING database of the World Cancer Research Fund International shows that many policies to restrict food promotion to children have been implemented to date and that the number of evaluation studies is increasing [55]. Summary of evidence on the effect of such policies and how they address socio-economic inequalities is advisable.

The study quality at primary study level as reported by review authors was mainly low to medium, reflecting that modelling studies form a large part of the evidence base. The quantity and quality of the evidence base would benefit from an increased number of empirical studies with integrated inequality perspectives. This would facilitate better understanding of how the effects of policies may vary with different levels of SEP, and to prioritize those policies with less chance of widening socioeconomic inequalities.

### Strengths and limitations

This study has several strengths. First, it applied a robust methodology to identify relevant systematic reviews that covered a range of food environment policies across the general population. We used the acknowledged Food-EPI framework to identify and analyze discreet policy areas of which there is considerable interest. We undertook quality evaluation using an acknowledged tool, the AMSTAR 2, to give a conservative assessment of our level of trust in the review findings. By using this methodology, we have provided an overview of the state of the evidence base.

Our review also has several limitations. Firstly, we only considered literature published in English, with the possibility that our review underrepresents studies from low- and middle-income countries. Second, we included reviews without an overall inequality focus to avoid missing reviews that were relevant but did not follow stringent reporting standards. As a result of this, three of our included reviews contributed with relatively limited content. Third, for some policy domains there was a marked overlap between primary studies in the included reviews. To limit duplication of findings we strived to report results as transparently as possible. Fourth, our searches were conducted in 2019 with search alerts revised in 2020, leaving a gap between the end of search and publication date. We cannot rule out that relevant systematic reviews may have been published during that time.

Another limitation of our study relates to the quality of included systematic literature reviews and the underlying primary studies. Most of the literature reviews in our umbrella review were considered low quality, with many of the reviews failing to meet several of the critical quality criteria of the AMSTAR 2. We often found descriptions of the included primary studies vague, which may be an intrinsic limitation of systematic literature reviews as there may be limited space to fully describe their included studies.

Lastly, reviews such as ours are designed to assess the inequality effects of discreet policies on diet-related outcomes. However, policies do not exist or operate in a vacuum. Indeed, dietary behaviors are impacted by complex and interwoven systems operating on many levels from individual to systems, where policy is only one out of many driving forces that interact with each other [56]. Over the last years, scholars have explored using system dynamics to understand the impact of a range of determinants on diet-related outcomes, including how determinants and other contextual factors interact and impact each other [57]. While it is important to consider the potential equity effects of each given policy, it is also important to take into account that not one single policy is a silver bullet to tackle unhealthy food environments, obesity or socioeconomic inequalities in diets and that comprehensive policy approaches are needed. Using systems-based longitudinal approaches in policy evaluation, also in the context of socioeconomic inequalities in diets, is recommended.

## Conclusions

Unhealthy diets and diet-related risk factors account for a large part of mortality and morbidity globally. There is an urgent need for policy measures to reduce these and their related socioeconomic inequalities. A robust evidence base on the effectiveness of food environment policies on socioeconomic inequalities could facilitate measures that are more likely to reduce the gap between socioeconomic groups. Our umbrella review shows that current research, at least at systematic review level, largely fails to provide this much-needed data. Tentative conclusions that can be drawn from our umbrella review support current policy recommendations that food pricing measures, in particular imposing taxes on unhealthy foods, can contribute to reducing socioeconomic inequalities in diets. Further research to assess the equity potential of policy domains presently underexplored such as food promotion, food composition, food labelling and food provision is highly recommended.

## Supplementary Information

**Additional file 1.** PRISMA Checklist. Table explaining where PRISMA items are reported in the manuscript, if applicable.

**Additional file 2.** Medline search strategy. Search string as performed in Medline.

**Additional file 3.** Full text screening guide. Detailed guidance with exclusion and inclusion criteria used in the full text screening stage.

**Additional file 4.** Example data extraction form. Table showing the different items that were extracted for the umbrella review.

**Additional file 5.** Revised AMSTAR 2. Table showing all AMSTAR items and how certain items were revised before quality appraisal.

**Additional file 6.** AMSTAR 2 assessment. Table showing the quality assessment per AMSTAR item.

**Additional file 7.** List of excluded articles. A list of the articles that were excluded at full text screening, with five different exclusion reasons.

**Additional file 8.** Study characteristics. Table describing study characteristics and key findings of included systematic reviews.

**Additional file 9.** List of primary studies reported in reviews. A list showing all primary studies that form the evidence base of the included systematic reviews included in the umbrella review, also showing what primary studies have been reported in more than one review.

## Data Availability

The datasets used and/or analyzed during the current study are available from the corresponding author on reasonable request.

## Abbreviations

AMSTAR 2: Assessing the Methodological Quality of Systematic Reviews quality assessment checklist
F&V: Fruit and vegetables
Food-EPI: The Healthy Food Environment Policy Index
FOP: Front of pack
INFORMAS: Network for Food and Obesity/NCDs Research, Monitoring and Action Support
NCDs: Non-communicable diseases
PRISMA: Preferred Reporting Items for Systematic Reviews and Meta-Analyses
SEP: Socioeconomic position
SNAP: Supplemental Nutrition Assistance Program
WHO: World Health Organization
WIC: Special Supplemental Nutrition Program for Women, Infants, and Children
