# Supplementary material for: The potential of food environment policies to reduce socioeconomic inequalities in diets and to improve healthy diets among lower socioeconomic groups: an umbrella review
Source: BMC Public Health. 2022 Mar 4;22:433. doi: 10.1186/s12889-022-12827-4 (PMC8895543; doi:10.1186/s12889-022-12827-4)
Supplement: Supplementary file 2 — Additional file 2. Medline search strategy. Search string as performed in Medline. [file 12889_2022_12827_MOESM2_ESM.docx]

# Additional file 2. Medline search strategy

1. government programs/ or policy/
2. public policy/ or health policy/ or nutrition policy/
3. Government Regulation/
4. Food Labeling/
5. Taxes/
6. (policy or policies).mp.
7. ((Government or public) adj3 (regulat* or program* or plan* or strateg* or tax* or levy or levies)).mp.
8. 1 or 2 or 3 or 4 or 5 or 6 or 7
9. Food/
10. feeding behavior/ or food preferences/
11. Health Behavior/
12. feeding behavior/ or food preferences/
13. Fast Foods/
14. diet/ or healthy diet/
15. Eating/
16. Obesity/
17. Nutritional status/
18. Noncommunicable Diseases/
19. sugars/ or dietary sugars/
20. Consumer Behavior/
21. (Food or diet* or eat or eating or nutrition* or sugar).mp.
22. 9 or 10 or 11 or 12 or 13 or 14 or 15 or 16 or 17 or 18 or 19 or 20 or 21
23. socioeconomic factors/ or economic status/ or poverty/ or poverty areas/ or social class/
24. Health Status Disparities/
25. Health Equity/
26. Income/
27. Poverty/
28. living standard.mp.
29. ((high or low) adj income).mp.
30. ((poverty or deprivation or low income) adj area*).mp.
31. socioeconomic.mp.
32. ((social* or health or economic or education* or occupation*) adj3 (disparit* or equity or equities or inequity or inequities or inequalit*)).mp.
33. 23 or 24 or 25 or 26 or 27 or 28 or 29 or 30 or 31 or 32
34. 8 and 22 and 33
35. "Systematic Review"/ or "Review"/
36. Meta-Analysis/
37. (systematic review* or metaanal* or meta-anal*).mp.
38. 35 or 36 or 37
39. 34 and 38
40. limit 39 to yr="2004 -Current"
