# Supplementary material for: The potential of food environment policies to reduce socioeconomic inequalities in diets and to improve healthy diets among lower socioeconomic groups: an umbrella review
Source: BMC Public Health. 2022 Mar 4;22:433. doi: 10.1186/s12889-022-12827-4 (PMC8895543; doi:10.1186/s12889-022-12827-4)
Supplement: Supplementary file 3 — Additional file 3. Full text screening guide. Detailed guidance with exclusion and inclusion criteria used in the full text screening stage. [file 12889_2022_12827_MOESM3_ESM.docx]

# Additional file 3. Full text screening guide

**Inclusion/exclusion criteria**

All the criteria below must be passed in order to be included. Use the order provided to assign exclusion reason in Rayyan.

1. **Study design:** Only include systematic literature reviews, scoping reviews and umbrella reviews. The studies included in systematic reviews can be of all *quantitative* study designs.
2. **Exposure/intervention:** Reviews that concern *the effect of food environment policies or interventions that test/explore such policies***.** Refer to the Food-EPI policy areas listed in the table below.

- **Exclude reviews that focus on other policies** (e.g. breastfeeding, alcohol, micronutrients, under-nutrition) OR interventions that look at education measures etc. If a paper includes both policy and education interventions, it can be included (and only relevant data retrieved subsequently).
- Exclude papers that look at methodology development etc.

1. **Socioeconomic inequality:** Only include reviews that analyze differential outcomes according to SEP OR that look at policies/interventions that are specifically targeted at low SEP communities.
   - SEP can be measured by education, income, area deprivation but NOT by ethnicity or rural setting.
   - To be included, inequality must either be explicitly framed in e.g. title, introduction, results and discussion OR, if not stated in introduction or methods, must emerge in results and discussion. Papers will be categorized according to its inequality focus later.
2. **Outcome:** Only include papers that look at outcomes:

- I: directly related to diet, including dietary intake; obesity and changes in BMI or weight
- II: intermediate dietary outcomes such as food purchases measured by household expenditure.
- We *exclude* papers that look at other outcomes, like access to health care; food knowledge, preferences etc.

| **Food environment policy areas** | **Explanation** | **Examples** |
| --- | --- | --- |
| **Food composition** | Policies to improve the nutritional quality of the food supply, in particular processed foods and out-of-home meals. | Mandatory or voluntary policies on the composition of foods sold from food stores or food service outlets. E.G: salt reduction; regulations banning the use of industrial trans fats |
| **Food labelling** | Policies on food labelling to help consumers make healthier, informed choices. | Standards for ingredient lists/nutrient declarations; regulatory systems for health and nutrition claims, evidence-based front-of-pack labelling schemes, menu labelling |
| **Food marketing to children and adolescents** | Policies that restrict promotion of unhealthy food to children and adolescents in different media and settings. | Policies that restrict advertising for unhealthy foods targeted to children in broadcast or digital media or other media like cinemas, print etc; ; on food packaging, and in schools. |
| **Food prices** | Economic tools to stimulate healthy food purchases and disincentive unhealthy food purchases | Reduced taxes on healthy foods; increased taxes on unhealthy foods; subsidies and programmes favour healthy foods;  food-related income programs are for healthy foods. |
| **Food provision** | Policies to promote healthy foods in schools and other public settings | Nutrition standards for schools and other public settings; healthy procurement policies for public settings; support systems for public and private settings |
| **Food retail** | Policies to improve neighborhood food environments so that healthy foods are available and accessible | Policies (e.g. zoning laws) that restrict the establishments of food outlets that primarily sell unhealthy foods; policies that encourage establishment of stores/outlets that sell healthier foods; support systems for stores and food service outlets to make them offer healthier foods. |
