# Supplementary material for: The potential of food environment policies to reduce socioeconomic inequalities in diets and to improve healthy diets among lower socioeconomic groups: an umbrella review
Source: BMC Public Health. 2022 Mar 4;22:433. doi: 10.1186/s12889-022-12827-4 (PMC8895543; doi:10.1186/s12889-022-12827-4)
Supplement: Supplementary file 4 — Additional file 4. Example data extraction form. Table showing the different items that were extracted for the umbrella review. [file 12889_2022_12827_MOESM4_ESM.docx]

# Additional file 4. Example data extraction form

| **Part 1. General information** | |
| --- | --- |
| **Item name** | **Explanation/**  **example** |
| **Reference/ year**  **(search time frame)** | Reference shortened and publication year, e.g. Hansen et al, 2016  (in brackets: the search time frame of the review as reported by review authors, e.g. search time frame 2003-2013) |
| **Aim of study** | E.g. “To assess the effects of food labelling on health-related outcomes and if effects vary by different characteristics” |
| **Included study designs in review** | Study designs as described by the review authors OR described based on included studies. E.g. quasi-experimental designs, cluster RCTs, modelling studies |
| **Setting/**  **Country/ies** | The settings of the included studies, either based on inclusion criteria or on data from the included studies.  E.g. Western countries; Low-income countries; U.S.A and UK |
| **Population** | Population as defined in inclusion criteria OR described based on data from included studies.  E.g. children, adults etc. |
| **Quality** | If possible, the overall level of quality as reported by the systematic review authors is briefly described, and quality assessment tools applied |
| **Focus on inequality** | Describes whether inequality is a main focus of the review and how SES is addressed |
| **Outcomes** | E.g. food intake, purchase, BMI |

| **Part 2. Information related to food environment policies and results** | |
| --- | --- |
| 1. **Policy domain(s)** | In this cell, the intervention(s) from each review is categorized according to the corresponding Food-EPI policy domains. Interventions are categorized as one or more of these domains:  **Food composition^1^**/ **Food labelling^2^ / Food promotion^3^/ Food prices^4^/ Food provision^5^/ Food in retail^6^** |
| 1. **n(N)** | How many primary studies out of the total number of studies in each review that is relevant for the umbrella review (meeting inclusion criteria in terms of exposure [policy/intervention], outcomes and SEP (e.g. 6/11). If one review encompasses several policy categories, the rate is given for each category (e.g. 3/11 for food labelling AND 4/11 for food prices) |
| 1. **Intervention components** | A description of the policies/intervention(s) based on data from the review. |
| 1. **Comments** | Any provided relevant information that may be useful to interpret results |
| 1. **Summary of results** | Description of results in absolute and relative terms for the SEP groups in the study, based on data from the reviews. |
| 1. **Direction of results: Impact/effectiveness** | If possible, description of effect of policies as negative, neutral or positive on socioeconomic inequality in diets based on the majority of effects found in each primary study, OR a summary of the overall effect on low SEP groups. |

^1^*Rules for the content of nutrients in packaged foods or foods sold in restaurants;*^2^ *Rules for ingredient lists/FOP labels on packaged foods, menus;*^3^*Restrictions on marketing in broadcast, digital, “traditional” media (print, cinema, outdoor etc.), in schools and other places children gather, and on food packaging*

^4^ *Increased taxes on unhealthy foods or decreased taxes on healthy foods; subsidies are targeted for healthy foods, income support programs support healthy foods*

^5^*Rules for food served in schools/public settings/other settings/procurement policies;*^6^*Zoning laws for communities, guidelines and support for food shops and restaurants; ^7^Describe if the interventions had other aspects that are not related to Food-EPI*
