# Supplementary material for: The potential of food environment policies to reduce socioeconomic inequalities in diets and to improve healthy diets among lower socioeconomic groups: an umbrella review
Source: BMC Public Health. 2022 Mar 4;22:433. doi: 10.1186/s12889-022-12827-4 (PMC8895543; doi:10.1186/s12889-022-12827-4)
Supplement: Supplementary file 5 — Additional file 5. Revised AMSTAR 2. Table showing all AMSTAR items and how certain items were revised before quality appraisal. [file 12889_2022_12827_MOESM5_ESM.docx]

# Additional file 5. Revised AMSTAR-2

**Revised *AMSTAR 2: a critical appraisal tool for systematic reviews that include randomized or non-randomized studies of healthcare interventions, or both***

**Text set in green has been revised**

**[INSERT REFERENCE + YEAR HERE]**

| 1. **Did the research question and inclusion criteria for the review include the components of PICO?** | | |
| --- | --- | --- |
| **For yes (at least the first three)**  Population  Intervention/policy  Outcome  Comparator (i.e. SEP groups) | | Yes  No |
| Revision:   - Added: *(at least the first three)* - Added: *(i.e. SEP groups)* - Deleted from original item 1: optional, recommended item *Timeframe for follow up* | | |
| 1. **Did the report of the review contain an explicit statement that the review methods were established prior to the conduct of the review and did the report justify any significant deviations from the protocol?** | | |
| **For Partial Yes:**  The authors state that they had a written protocol or guide that included ALL the following:  review question(s)  a search strategy  inclusion/exclusion criteria  a risk of bias assessment | **For Yes:**  As for partial yes, plus the protocol should be registered and should also have specified:  a synthesis plan  justification for any deviations from the protocol, if applicable | Yes  Partial yes  No |
| Revision: Deleted: For Yes: *a meta-analysis/synthesis plan, if appropriate* | | |
| 1. **Did the review authors explain their selection of the study designs for inclusion in the review?** | | |
| For Yes, the review should satisfy ONE of the following:  Explanation for including only RCTs  OR Explanation for including only other study designs  OR Explanation for including both RCTs and other study designs | | Yes  No |
| Revision: used *Other study designs* rather than *NRSI* | | |
| 1. **Did the review authors use a comprehensive literature search strategy?** | | |
| **For Partial Yes** (all the following):  searched at least 2 databases (relevant to research question)  provided key word and/or search strategy  justified publication restrictions* | **For Yes**, should also have (all the following):  searched the reference lists / bibliographies of included studies  searched trial/study registries (if applicable)  included/consulted content experts in the field  where relevant, searched for grey literature  conducted search within 24 months of completion of the review | Yes  Partial yes  No |
| Revision: deleted from Partial yes: *(e.g. language) | | |
| 1. **Did the review authors perform study selection in duplicate?** | | |
| **For Yes**, either ONE of the following:  at least two reviewers independently agreed on selection of eligible studies and achieved consensus on which studies to include  OR two reviewers selected a sample of eligible studies and achieved good agreement (at least 80 percent), with the remainder selected by one reviewer. | | Yes  No |
| 1. **Did the review authors perform data extraction in duplicate?** | | |
| **For Yes**, either ONE of the following:  at least two reviewers achieved consensus on which data to extract from included studies  OR two reviewers extracted data from a sample of eligible studies and achieved good agreement (at least 80 percent), with the remainder extracted by one reviewer. | | Yes  No |
| 1. **Did the review authors provide a list of excluded studies and justify the exclusions?** | | |
| **For Partial Yes:**  provided a list of all potentially relevant studies that were read in full-text form but excluded from the review | **For Yes,** must also have:  Justified the exclusion from the review of each potentially relevant study | Yes  Partial Yes  No |
| 1. **Did the review authors describe the included studies in adequate detail?** | | |
| For Partial Yes (all the following)  described populations  described interventions/policies  described comparators *(if applicable)*  described outcomes  described research designs | For Yes, should also have ALL the following:  described population in detail  described intervention/policy in detail*  described comparator in detail*  described study’s setting  timeframe for follow-up | Yes  Partial yes  No |
| For Yes, when applicable, a “detailed description” of Population includes a description of how SEP is measured; “detailed description” of intervention/policy includes to describe any other intervention components that may have affected the results (e.g. a combination of policy and education). Setting = geographical | | |
| Revisions:   - for Partial yes: added *policies* and *(if applicable)* - for Yes: added *policy*, removed * *(including doses where relevant)* | | |
| 1. **Did the review authors use a satisfactory technique for assessing the risk of bias (RoB) in individual studies that were included in the review?** | | |
| For partial yes: at least two of the following:  confounding  sample selection bias  publication bias | For yes, must also have assessed:  methods used to ascertain exposures and outcomes, and  selection of the reported result from among multiple measurements or analyses of a specified outcome | Yes  Partial yes  No |
| Revision:   - removed item specific for RCTs - For Partial Yes, added *at least two* and the option for *publication bias* | | |
| **10. Did the review authors report on the sources of funding for the studies included in the review?** | | |
| For Yes  Must have reported on the sources of funding for individual studies included in the review. Note: Reporting that the reviewers looked for this information but it was not reported by study authors also qualifies | | Yes  No |
| **11. If meta-analysis was performed did the review authors use appropriate methods for statistical combination of results?** | | |
| **RCTs**  For Yes:  The authors justified combining the data in a meta-analysis  AND they used an appropriate weighted technique to combine study results and adjusted for heterogeneity if present.  AND investigated the causes of any heterogeneity | **For other study designs**  For Yes:  The authors justified combining the data in a meta-analysis  AND they used an appropriate weighted technique to combine study results, adjusting for heterogeneity if present  AND they statistically combined effect estimates from other study designs that were adjusted for confounding, rather than combining raw data, or justified combining raw data when adjusted effect estimates were not available  AND they reported separate summary estimates for RCTs and other study designsseparately when both were included in the review | Yes  No  No meta-analysis conducted |
| **12. If meta-analysis was performed, did the review authors assess the potential impact of RoB in individual studies on the results of the meta-analysis or other evidence synthesis?** | | |
| For Yes:  included only studies with low risk of bias  OR, if the pooled estimate was based on studies at variable RoB, the authors performed analyses to investigate possible impact of RoB on summary estimates of effect. | | Yes  No  No meta-analysis conducted |
| Revision: used *studies* rather than *RCTs* | |  |
| **13. Did the review authors account for RoB in individual studies when interpreting/ discussing the results of the review?** | | |
| For Yes:  included only studies with low risk of bias  OR provided a discussion of the likely impact of RoB on the results | | Yes  No |
| Revision:   - *Used studies* rather than *RCTs*. - Revised from the original text: *OR, if RCTs with moderate or high RoB, or NRSI were included the review provided a discussion of the likely impact of RoB on the results* | | |
| **14. Did the review authors provide a satisfactory explanation for, and discussion of, any heterogeneity observed in the results of the review?** | | |
| For Yes:  There was no significant heterogeneity in the results  OR if heterogeneity was present the authors performed an investigation of sources of any heterogeneity in the results and discussed the impact of this on the results of the review | | Yes  No |
| **15. If they performed quantitative synthesis did the review authors carry out an adequate investigation of publication bias (small study bias) and discuss its likely impact on the results of the review?** | | |
| For Yes:  performed graphical or statistical tests for publication bias and discussed the likelihood and magnitude of impact of publication bias | | Yes  No  No meta-analysis conducted |
| **16. Did the review authors report any potential sources of conflict of interest, including any funding they received for conducting the review?** | | |
| For Yes:  The authors reported no competing interests OR  The authors described their funding sources and how they managed potential conflicts of interest | | Yes  No |
