# Supplementary material for: The potential of food environment policies to reduce socioeconomic inequalities in diets and to improve healthy diets among lower socioeconomic groups: an umbrella review
Source: BMC Public Health. 2022 Mar 4;22:433. doi: 10.1186/s12889-022-12827-4 (PMC8895543; doi:10.1186/s12889-022-12827-4)
Supplement: Supplementary file 6 — Additional file 6. AMSTAR 2 assessment. Table showing the quality assessment per AMSTAR item. [file 12889_2022_12827_MOESM6_ESM.docx]

# Additional file 6. AMSTAR-2 assessment

| **Reference** | 1 | **2** | 3 | **4** | 5 | 6 | 7 | **8** | **9** | 10 | 11 | 12 | **13** | 14 | 15 | 16 | **OVERALL** |
| --- | --- | --- | --- | --- | --- | --- | --- | --- | --- | --- | --- | --- | --- | --- | --- | --- | --- |
| Abeykoon et al 2017 ^1^ | Y | **N** | Y | **PY** | N | N | N | **Y** | **PY** | N | NA | NA | **Y** | Y | NA | Y | Low |
| Andreyeva et al 2010^2^ | Y | **N** | Y | **PY** | Y | Y | N | **N** | **N** | N | NA | NA | **N** | N | NA | N | Critically low |
| Backholer et al 2016^3^ | Y | **PY** | Y | **PY** | Y | Y | N | **PY** | **N** | N | NA | NA | **N** | Y | NA | Y | Critically low |
| Black et al 2012^4^ | Y | **N** | Y | **PY** | Y | N | N | **Y** | **Y** | N | NA | NA | **Y** | Y | NA | Y | Low |
| Cuffey et al 2015^5^ | Y | **N** | Y | **PY** | N | N | N | **PY** | **N** | N | NA | NA | **Y** | Y | NA | Y | Critically low |
| Eyles et al 2012^6^ | Y | **N** | Y | **PY** | N | N | N | **Y** | **Y** | N | NA | NA | **Y** | Y | NA | Y | Low |
| Hartmann-  Boyce et al 2018^7^ | Y | **Y** | Y | **PY** | Y | Y | N | **Y** | **PY** | N | NA | NA | **N** | Y | NA | Y | Low |
| Hendry et al 2015^8^ | Y | **Y** | Y | **PY** | Y | Y | N | **PY** | **PY** | N | NA | NA | **Y** | Y | NA | N | Moderate |
| McGill et al 2015^9^ | Y | **N** | Y | **PY** | Y | Y | N | **Y** | **PY** | Y | NA | NA | **Y** | Y | NA | Y | Low |
| Nakhimovsky et al 2016^10^ | Y | **PY** | Y | **PY** | Y | Y | N | **Y** | **PY** | Y | NA | NA | **Y** | Y | NA | Y | High |
| Olstad et al 2016^11^ | Y | **Y** | Y | **PY** | Y | Y | N | **PY** | **PY** | N | NA | NA | **Y** | Y | NA | Y | Moderate |
| Olstad et al 2017^12^ | Y | **Y** | Y | **PY** | Y | Y | N | **Y** | **PY** | N | NA | NA | **Y** | Y | NA | Y | Moderate |
| Sarink et al 2016^13^ | Y | **N** | Y | **PY** | N | Y | N | **PY** | **PY** | N | NA | NA | **Y** | Y | NA | Y | Low |
| Schultz et al 2015^14^ | Y | **N** | N | **PY** | Y | Y | N | **PY** | **PY** | Y | NA | NA | **N** | N | NA | Y | Critically low |
| Thow et al 2010^15^ | Y | **N** | Y | **PY** | N | N | N | **PY** | **N** | N | NA | NA | **Y** | Y | NA | Y | Critically low |
| Thow et al 2014^16^ | Y | **N** | Y | **PY** | N | N | N | **PY** | **PY** | N | NA | NA | **Y** | N | NA | Y | Low |

Quality appraisal of included reviews including critical domains and overall confidence in the results. AMSTAR-2 includes the following items, where numbers set in **bold** are critical domains used to determine an overall assessment of confidence in the review results: (1) PICO components included in research question and inclusion criteria for the review. **(2)** A priori research design provided. (3) Selection of study designs explained. **(4)** Comprehensive search strategy used. (5) Study selection performed in duplicate. (6) Data extraction performed in duplicate. (7) List of excluded studies provided. **(8)** Included studies described in detail. **(9)** Satisfactory assessment of risk of bias in individual studies. (10) Source of funding for individual studies reported; (11) appropriate methods for statistical combination of results (in meta-analysis); (12) Risk of bias in individual studies assessed in results of meta-analysis); **(13)** Risk of bias accounted for in interpretation of results in the review; (14) Explanation of heterogeneity in review results explained. (15) Investigation of publication bias (in meta-analysis). (16) Funding and conflict of interest for review reported.
