# Supplementary material for: The potential of food environment policies to reduce socioeconomic inequalities in diets and to improve healthy diets among lower socioeconomic groups: an umbrella review
Source: BMC Public Health. 2022 Mar 4;22:433. doi: 10.1186/s12889-022-12827-4 (PMC8895543; doi:10.1186/s12889-022-12827-4)
Supplement: Supplementary file 7 — Additional file 7. List of excluded articles. A list of the articles that were excluded at full text screening, with five different exclusion reasons. [file 12889_2022_12827_MOESM7_ESM.docx]

# Additional file 7. List of excluded articles

The articles are listed according to the following exclusion reasons, in hierarchical order:

1. Wrong study design (not systematic literature review or not scientific article)
2. Wrong exposure (article did not assess policy, or included policies were out of scope)
3. No focus on inequality
4. Not sufficient focus on inequality (did not explicitly report differential effects of the policy intervention on different socioeconomic groups or in low-income groups)
5. Other (reasons provided for each article)

## Wrong study design (n=45)

Afshin A, Penalvo J, Del Gobbo L, Kashaf M, Micha R, Morrish K, et al. CVD Prevention Through Policy: a Review of Mass Media, Food/Menu Labeling, Taxation/Subsidies, Built Environment, School Procurement, Worksite Wellness, and Marketing Standards to Improve Diet. Current Cardiology Reports. 2015;17(11):98.

Attree, P. (2005). "Low-income mothers, nutrition and health: a systematic review of qualitative evidence." Maternal & Child Nutrition 1(4): 227-240.

Attree, P. (2006). "A critical analysis of UK public health policies in relation to diet and nutrition in low-income households." Maternal & Child Nutrition 2(2): 67-78.

Backholer, K., et al. (2019). "Food and Beverage Price Promotions: an Untapped Policy Target for Improving Population Diets and Health." Current Nutrition Reports 8(3): 250-255.

Backholer, K., et al. (2014). "The effect of a sugar sweetened beverage tax among different socioeconomic groups: A systematic review." Obesity Research and Clinical Practice 1: 4-5. (*Conference abstract)*

Bambra, C. L., et al. (2015). "How effective are interventions at reducing socioeconomic inequalities in obesity among children and adults? Two systematic reviews." Public health research. 3 (1). *(This is a report)*

Bambra, C. L., et al. (2012). "Tackling inequalities in obesity: a protocol for a systematic review of the effectiveness of public health interventions at reducing socioeconomic inequalities in obesity amongst children." Systematic Reviews 1: 16.

Barton, R. L. and K. Whitehead (2008). "A review of community based healthy eating interventions." Journal of Human Nutrition & Dietetics 21(4): 378-379.

Bes-Rastrollo, M., et al. (2016). "Impact of sugars and sugar taxation on body weight control: A comprehensive literature review." Obesity (Silver Spring, Md.) 24(7): 1410-1426.

Bonell, C., et al. (2013). Systematic review of the effects of schools and school environment interventions on health: evidence mapping and synthesis. *(This is a report)*

Bowen, D. J., et al. (2015). "Identifying the effects of environmental and policy change interventions on healthy eating." Annual Review of Public Health 36: 289-306.

Brambila-Macias, J., et al. (2011). "Policy interventions to promote healthy eating: a review of what works, what does not, and what is promising." Food & Nutrition Bulletin 32(4): 365-375.

Brug, J., et al. (2006). "Revisiting Kurt Lewin - How to gain insight into environmental correlates of obesogenic behaviors." American Journal of Preventive Medicine 31(6): 525-529.

Cairns, G. (2019). "A critical review of evidence on the sociocultural impacts of food marketing and policy implications." Appetite 136: 193-207.

César Patricio, B. and T. Luis (2007). Do we know what works? A systematic review of impact evaluations of social programs in Latin America and the Caribbean. *(This is a working paper)*

Cobb, L. K., et al. (2015). "The local food environment and obesity: A systematic review." Circulation. Conference: American Heart Association's Epidemiology and Prevention/Lifestyle and Cardiometabolic Health 131. *(Conference abstract)*

Colón-Ramos, U., et al. (2014). "Impact of WHO recommendations to eliminate industrial trans-fatty acids from the food supply in Latin America and the Caribbean." Health Policy & Planning 29(5): 529-541.

Coufopoulos, A., et al. (2012). "Interventions to improve nutrition and nutrition related health amongst homeless mothers and their children: A systematic review." Proceedings of the Nutrition Society. Conference: Summer Meeting of the Nutrition Society Hosted by the Irish Section 71.

Dixon, B. N., et al. (2019). "A Social-ecological Review of the Rural versus Urban Obesity Disparity." Health Behavior and Policy Review 6(4): 378-394.

Evans, C. E., et al. (2015). "School-Based Interventions to Reduce Obesity Risk in Children in High- and Middle-Income Countries." Advances in Food & Nutrition Research 76: 29-77.

Faulkner, G. E., et al. (2011). "Economic instruments for obesity prevention: results of a scoping review and modified Delphi survey." International Journal of Behavioral Nutrition & Physical Activity 8: 109.

Federici, C., et al. (2016). "The effects of food prices on weight outcomes. A systematic review of the moderating effects of income and initial BMI." Value in Health 19: A454-A455.

Gelfer, M., et al. (2014). "Calls for restricting the marketing of unhealthy foods to children ignored by policy makers: What can we do?" Canadian Family Physician 60(11): 969-971 and 978-980.

Gortmaker, S. L., et al. (2015). "Three interventions that reduce childhood obesity are projected to save more than they cost to implement." Health Affairs 34(11): 1932-1939.

Grier, S. A. and S. K. Kumanyika (2008). "The Context for Choice: Health Implications of Targeted Food and Beverage Marketing to African Americans." American Journal of Public Health 98(9): 1616-1629.

Hannon, E. and B. Learner (2016). "The impact of breakfast programs on health and wellbeing in western europe." Revista Espanola de Nutricion Humana y Dietetica 20: 541-542.

Hawkes, C. (2005). "Self-regulation of food advertising: What it can, could and cannot do to discourage unhealthy eating habits among children." Nutrition Bulletin 30(4): 374-382.

Hawley, K. L., et al. (2013). "The science on front-of-package food labels." Public Health Nutrition 16(3): 430-439.

Knai C, Petticrew M, Durand MA, Eastmure E, James L, Mehrotra A, et al. Has a public–private partnership resulted in action on healthier diets in England? An analysis of the Public Health Responsibility Deal food pledges. Food Policy. 2015;54:1-10.

Kraak, V. I., et al. (2012). "Government and school progress to promote a healthful diet to American children and adolescents: a comprehensive review of the available evidence." American Journal of Preventive Medicine 42(3): 250-262.

Kumanyika SK, Swank M, Stachecki J, Whitt-Glover MC, Brennan LK. Examining the evidence for policy and environmental strategies to prevent childhood obesity in black communities: new directions and next steps. Obesity reviews : an official journal of the International Association for the Study of Obesity. 2014;15:177-203.

Magnusson, R. S. (2008). "What's law got to do with it Part 2: Legal strategies for healthier nutrition and obesity prevention." Australia and New Zealand Health Policy 5(11).

Micha, R., et al. (2016). "Effectiveness of school procurement policies for improving dietary behaviors: A systematic review and meta-analysis." Circulation. Conference: American Heart Association's Epidemiology and Prevention/Lifestyle and Cardiometabolic Health 133.

Nakhimovsky, S., et al. (2017). "The effectiveness of using taxes on sugar-sweetened beverages to reduce obesity in middle income countries: A systematic review." Annals of Global Health: 545. *(This is a conference abstract/poster presentation)*

Oliver, S., et al. (2008). Health promotion, inequalities and young people's health: a systematic review of research. *(This is a report)*

Orton, L., et al. (2013). "Using the "4Ps" marketing approach to evaluate healthy food policies: A rapid scoping review." European Journal of Epidemiology 1: S35. *(This is a conference abstract/poster presentation)*

Orton, L., et al. (2013). "Assessing the potential effect of healthy eating policy interventions on socioeconomic inequalities: Systematic review." European Journal of Epidemiology 1: S81. *(This is a conference abstract/poster presentation)*

Pillaca-Medina, S. and P. N. Chavez-Dulanto (2017). "How effective and efficient are social programs on food and nutritional security?: The case of Peru: A review How effective and efficient are social programs on food and nutritional security? : The case of Peru: A review PILLACA-MEDINA and CHAVEZ-DULANTO." Food and Energy Security 6(4).

Powell, L. M. and F. J. Chaloupka (2009). "Food prices and obesity: evidence and policy implications for taxes and subsidies." The Milbank quarterly 87(1): 229-257.

Priest, N., et al. (2008). "Policy interventions implemented through sporting organisations for promoting healthy behaviour change." Cochrane Database of Systematic Reviews(3).

Rodriguez-Fern, et al. (2013). "Current salt reduction policies across gradients of inequality-adjusted human development in the WHO European region: minding the gaps." Public Health Nutrition 17(8): 1894-1904.

Ronit, K. and J. D. Jensen (2014). "Obesity and industry self-regulation of food and beverage marketing: a literature review." European Journal of Clinical Nutrition 68(7): 753-759.

Saguil, A. and M. Stephens (2012). "Interventions to Prevent Childhood Obesity." American Family Physician 86(1): 30-32.

Thomson, K., et al. (2017). "The effects of public health policies on health inequalities: A review of reviews." The Lancet 390: S12. *(Meeting abstract)*

Wang Y, Wu Y, Wilson RF, Bleich S, Cheskin L, Weston C, et al. Childhood Obesity Prevention Programs: Comparative Effectiveness Review and Meta-Analysis2013 2013-06-None. *(Book)*

## Wrong exposure (n=48)

Avery A, Bostock L, McCullough F. A systematic review investigating interventions that can help reduce consumption of sugar‐sweetened beverages in children leading to changes in body fatness. J Hum Nutr Diet. 2015;28(s1):52-64.

AlMarzooqi, M. A. and M. C. Nagy (2011). "Childhood Obesity Intervention Programs: A Systematic Review." Life Science Journal-Acta Zhengzhou University Overseas Edition 8(4): 45-60.

Beauchamp A, Backholer K, Magliano D, Peeters A. The effect of obesity prevention interventions according to socioeconomic position: a systematic review. Obesity Reviews. 2014;15(7):541-54. (Only multicomponent studies makes it difficult to extract data on policy)

Brennan LK, Brownson RC, Orleans CT. Childhood obesity policy research and practice: evidence for policy and environmental strategies. American journal of preventive medicine. 2014;46(1):e1-16.

Carter, M. A., et al. (2012). "Availability and marketing of food and beverages to children through sports settings: a systematic review." Public Health Nutrition 15(8): 1373-1379.

Dangour AD, Hawkesworth S, Shankar B, Watson L, Srinivasan CS, Morgan EH, et al. Can nutrition be promoted through agriculture-led food price policies? A systematic review. BMJ open. 2013;3(6).

Darmon N, Drewnowski A. Contribution of food prices and diet cost to socioeconomic disparities in diet quality and health: a systematic review and analysis. Nutrition Reviews. 2015;73(10):643-60. *(The study does not assess policy)*

Dunton, G. F., et al. (2009). "Physical environmental correlates of childhood obesity: a systematic review." Obesity Reviews 10(4): 393-402.

Engler-Stringer, R., et al. (2019). "An Examination of Failed Grocery Store Interventions in Former Food Deserts." Health Education & Behavior 46(5): 749-754.

Gustafson, A., et al. (2012). "Measures of the Consumer Food Store Environment: A Systematic Review of the Evidence 2000-2011." Journal of Community Health 37(4): 897-911.

Harding, T. and J. Oetzel (2019). "Implementation effectiveness of health interventions for indigenous communities: a systematic review." Implementation science : IS 14(1): 76.

Hillier-Brown, F. C., et al. (2014). "A systematic review of the effectiveness of individual, community and societal level interventions at reducing socioeconomic inequalities in obesity amongst children." BMC Public Health 14: 834.

Hilmers, A., et al. (2012). "Neighborhood disparities in access to healthy foods and their effects on environmental justice." American Journal of Public Health 102(9): 1644-1654.

Hollands, G. J., et al. (2015). "Portion, package or tableware size for changing selection and consumption of food, alcohol and tobacco." Cochrane Database of Systematic Reviews (9).

Iacovou, M., et al. (2012). "Social health and nutrition impacts of community kitchens: a systematic review." Public Health Nutrition 16(3): 535-543.

Jourdan, D., et al. (2016). "The involvement of young people in school- and community-based noncommunicable disease prevention interventions: a scoping review of designs and outcomes." BMC Public Health 16(1): 1123.

Knai, C., et al. (2006). "Getting children to eat more fruit and vegetables: A systematic review." Preventive Medicine 42(2): 85-95.

Labonté, M., et al. (2018). "Nutrient Profile Models with Applications in Government-Led Nutrition Policies Aimed at Health Promotion and Noncommunicable Disease Prevention: A Systematic Review." Advances in nutrition (Bethesda, Md.) 9(6): 741-788.

Labonte, M. E., et al. (2017). "Global evidence on nutrient profile models with applications in government-led nutrition policies aimed at health promotion and noncommunicable disease prevention: A systematic review." Annals of Nutrition and Metabolism 71: 718.

Langford R, Bonell CP, Jones HE, Pouliou T, Murphy SM, Waters E, et al. The WHO Health Promoting School framework for improving the health and well-being of students and their academic achievement. Cochrane Database of Systematic Reviews. 2014(4).

Lee, J. H., et al. (2011). "Influence of food cost on diet quality and risk factors for chronic disease: A systematic review." Nutrition & Dietetics 68(4): 248-261.

Lobelo, F., et al. (2012). "School-based programs aimed at the prevention and treatment of obesity: evidence-based interventions for youth in Latin America." Journal of School Health 83(9): 668-677.

Luckner, H., et al. (2012). "Effectiveness of interventions to promote healthy weight in general populations of children and adults: a meta-analysis." European Journal of Public Health 22(4): 491-497.

Luybli, M., et al. (2019). "School-Based Interventions in Low Socioeconomic Settings to Reduce Obesity Outcomes among Preschoolers: A Scoping Review." Nutrients 11(7).

Matwiejczyk L, Mehta K, Scott J, Tonkin E, Coveney J. Characteristics of effective interventions promoting healthy eating for pre-schoolers in childcare settings: An umbrella review. Nutrients. 2018;10(293).

Mayen, A. L., et al. (2016). "Interventions promoting healthy eating as a tool for reducing social inequalities in diet in low- and middle-income countries: a systematic review." International Journal for Equity in Health 15.

Mazarello Paes, V., et al. (2015). "Determinants of sugar-sweetened beverage consumption in young children: a systematic review." Obesity reviews: an official journal of the International Association for the Study of Obesity 16(11): 903-913.

McIsaac, J. L. D., et al. (2019). "Factors Influencing the Implementation of Nutrition Policies in Schools: A Scoping Review." Health Education & Behavior 46(2): 224-250.

McKinnon, R. A., et al. (2016). "Obesity-Related Policy/Environmental Interventions: A Systematic Review of Economic Analyses." American Journal of Preventive Medicine 50(4): 543-549. *(This article assesses the wrong outcome)*

Minaker, L. M., et al. (2016). "Retail food environments research in Canada: A scoping review." Canadian journal of public health = Revue canadienne de sante publique 107: 5344.

Nguyen, M. T. T., et al. (2019). "A Systematic Review on the Effects of Personalized Price Promotions for Food Products." Journal of Food Products Marketing 25(3): 257-275.

Novakovic, R., et al. (2011). "Systematic review: Socioeconomic and cultural determinants of low micronutrient intake and status within EURRECA Network." Annals of Nutrition and Metabolism 3: 403-404.

Oldroyd, J., et al. (2008). "The effectiveness of nutrition interventions on dietary outcomes by relative social disadvantage: a systematic review." Journal of Epidemiology & Community Health 62(7): 573-579.

Pega, F., et al. (2017). "Unconditional cash transfers for reducing poverty and vulnerabilities: Effect on use of health services and health outcomes in low- and middle-income countries." Cochrane Database of Systematic Reviews 2017(11).

Pullar, J., et al. (2018). "The impact of poverty reduction and development interventions on noncommunicable diseases and their behavioural risk factors in low and lower-middle income countries: A systematic review." PLoS ONE 13.

Schröders, J., et al. (2017). "How is Indonesia coping with its epidemic of chronic noncommunicable diseases? A systematic review with meta-analysis." PLoS ONE 12(6): e0179186.

Sebastian-Ponce, M. I., et al. (2014). "Consumer reaction to information on the labels of genetically modified food." Rev. saúde pública 48(1): 154-169.

Sikorski, C., et al. (2011). "The stigma of obesity in the general public and its implications for public health - a systematic review." BMC Public Health 11(1): 661.

Silveira, J. A. C., et al. (2011). "Effectiveness of school-based nutrition education interventions to prevent and reduce excessive weight gain in children and adolescents: a systematic review." Jornal De Pediatria 87(5): 382-392.

Singh, K., et al. (2018). "Cost-effectiveness of interventions to control cardiovascular diseases and diabetes mellitus in South Asia: a systematic review." BMJ Open 8(4): e017809.

Tovar A, Renzaho AM, Guerrero AD, Mena N, Ayala GX. A Systematic Review of Obesity Prevention Intervention Studies among Immigrant Populations in the US. Current obesity reports. 2014;3(2):206-22.

Vargas CM, Stines EM, Granado HS. Health-equity issues related to childhood obesity: a scoping review. Journal of Public Health Dentistry. 2017;77:S32-S42.

Vitolins MZ, Crandall S, Miller D, Ip E, Marion G, Spangler JG. Obesity Educational Interventions in U.S. Medical Schools: A Systematic Review and Identified Gaps. Teaching and Learning in Medicine. 2012;24(3):267-72.

Volger S, Radler DR, Rothpletz-Puglia P. Early childhood obesity prevention efforts through a life course health development perspective: A scoping review. PLoS ONE. 2018;13.

Walker RE, Keane CR, Burke JG. Disparities and access to healthy food in the United States: A review of food deserts literature. Health and Place. 2010;16(5):876-84.

Wolfenden L, Goldman S, Stacey FG, Grady A, Kingsl, M, et al. Strategies to improve the implementation of workplace‐based policies or practices targeting tobacco, alcohol, diet, physical activity and obesity. Cochrane Database of Systematic Reviews. 2018(11).

Wolfenden L, Jones J, Williams CM, Finch M, Wyse RJ, Kingsl, et al. Strategies to improve the implementation of healthy eating, physical activity and obesity prevention policies, practices or programmes within childcare services. Cochrane Database of Systematic Reviews. 2016;10(10):CD011779.

Wolfenden L, Nathan NK, Sutherl, R, Yoong SL, Hodder RK, et al. Strategies for enhancing the implementation of school-based policies or practices targeting risk factors for chronic disease. Cochrane Database of Systematic Reviews. 2017(11).

## No focus on inequality (n=45)

Alagiyawanna, A., et al. (2015). "Studying the consumption and health outcomes of fiscal interventions (taxes and subsidies) on food and beverages in countries of different income classifications; a systematic review." BMC Public Health 15: 887.

Anastasiou, K., et al. (2019). "The relationship between food label use and dietary intake in adults: A systematic review." Appetite 138: 280-291.

Afshin A, Peñalvo JL, Del Gobbo L, Silva J, Michaelson M, O'Flaherty M, et al. The prospective impact of food pricing on improving dietary consumption: A systematic review and meta-analysis. PLoS ONE. 2017;12(3):1-18.

Bambra, C., et al. (2010). "Tackling the wider social determinants of health and health inequalities: Evidence from systematic reviews." Journal of Epidemiology and Community Health 64(4): 284-291. *(This review of reviews focused on health inequality but the included review that assessed relevant policies did not assess SEP)*

Bergallo, P., et al. (2018). "Regulatory initiatives to reduce sugar-sweetened beverages (SSBs) in Latin America." PLoS ONE 13(10): 1-17.

Bramante, C. T., et al. (2019). "Systematic Review of Natural Experiments for Childhood Obesity Prevention and Control." American Journal of Preventive Medicine 56(1): 147-158.

Calancie L, Leeman J, Jilcott Pitts SB, Khan LK, Fleischhacker S, Evenson KR, et al. Nutrition-related policy and environmental strategies to prevent obesity in rural communities: a systematic review of the literature, 2002-2013. Preventing chronic disease. 2015;12:E57.

Castro, I. A., et al. (2018). "Customer Purchase Intentions and Choice in Food Retail Environments: A Scoping Review." International Journal of Environmental Research and Public Health 15(11).

Cecchini, M. and L. Warin (2016). "Impact of food labelling systems on food choices and eating behaviours: a systematic review and meta-analysis of randomized studies." Obesity Reviews 17(3): 201-210.

Chambers, S. A., et al. (2015). "Reducing the volume, exposure and negative impacts of advertising for foods high in fat, sugar and salt to children: A systematic review of the evidence from statutory and self-regulatory actions and educational measures." Preventive Medicine 75: 32-43.

Chriqui, J. F., et al. (2014). "Influence of school competitive food and beverage policies on obesity, consumption, and availability: a systematic review." JAMA pediatrics 168(3): 279-286.

Colley, P., et al. (2018). "The Impact of Canadian School Food Programs on Children's Nutrition and Health: A Systematic Review." Canadian journal of dietetic practice and research: a publication of Dietitians of Canada = Revue canadienne de la pratique et de la recherche en dietetique : une publication des Dietetistes du Canada 80(2): 1-8.

Cornelsen, L., et al. (2014). "What happens to patterns of food consumption when food prices change? Evidence from a systematic review and meta-analysis of food price elasticities globally." Health Economics (United Kingdom) 24(12): 1548-1559.

Downs, S. M., et al. (2013). "The effectiveness of policies for reducing dietary trans fat: a systematic review of the evidence." Bulletin of the World Health Organization 91(4): 262-269H.

Driessen, C. E., et al. (2014). "Effect of changes to the school food environment on eating behaviours and/or body weight in children: a systematic review." Obesity Reviews 15(12): 968-982.

Engler-Stringer, R., et al. (2014). "The community and consumer food environment and children's diet: a systematic review." BMC Public Health 14(1): 1271-1292.

Fattore, G., et al. (2014). "Critical review of economic evaluation studies of interventions promoting low-fat diets." Nutrition Reviews 72(11): 691-706.

Fern, et al. (2016). "Influence of menu labeling on food choices in real-life settings: a systematic review." Nutrition Reviews 74(8): 534-548.

Fitzpatrick-Lewis D, Ciliska D, Peirson LJ, Warren RL, Fieldhouse P, Delgado-Noguera MF, et al. Enhancing nutritional environments through access to fruit and vegetables in schools and homes among children and youth: a systematic review. BMC research notes. 2014; 7:422.

Galbraith-Emami, S. and T. Lobstein (2013). "The impact of initiatives to limit the advertising of food and beverage products to children: a systematic review." Obesity Reviews 14(12): 960-974. *(This article also does not assess diet-related outcomes)*

Green, R., et al. (2013). "The effect of rising food prices on food consumption: systematic review with meta-regression." BMJ 346: f3703.

Harnack, L. J. and S. A. French (2008). "Effect of point-of-purchase calorie labeling on restaurant and cafeteria food choices: a review of the literature." The international journal of behavioral nutrition and physical activity 5: 51.

Hillier-Brown FC, Summerbell CD, Moore HJ, Routen A, Lake AA, Adams J, et al. The impact of interventions to promote healthier ready-to-eat meals (to eat in, to take away or to be delivered) sold by specific food outlets open to the general public: a systematic review. Obesity reviews: an official journal of the International Association for the Study of Obesity. 2016;18(2):227-46.

Hyseni, L., et al. (2017). "Systematic review of dietary salt reduction policies: Evidence for an effectiveness hierarchy?" PLoS ONE 12(5): e0177535.

Jaime, P. C. and K. Lock (2009). "Do school based food and nutrition policies improve diet and reduce obesity?" Preventive Medicine 48(1): 45-53.

Jensen, J. D., et al. (2011). "Economic incentives and nutritional behavior of children in the school setting: A systematic review." Nutrition Reviews 69(11): 660-674.

Johnson, B. J., et al. (2016). "Reducing discretionary food and beverage intake in early childhood: a systematic review within an ecological framework." Public Health Nutrition 19(9): 1684-1695.

Kahn-Marshall, J. L. and M. P. Gallant (2012). "Making healthy behaviors the easy choice for employees: a review of the literature on environmental and policy changes in worksite health promotion." Health education & behavior : the official publication of the Society for Public Health Education 39(6): 752-776.

Lambert EV, Parker W, Steyn NP, McHiza Z, De Villiers A. A review of school nutrition interventions globally as an evidence base for the development of the HealthKick programme in the Western Cape, South Africa: original research. South African Journal of Clinical Nutrition. 2009;22(3):145-52

Maniadakis N, Kapaki V, Damianidi L, Kourlaba G. A systematic review of the effectiveness of taxes on nonalcoholic beverages and high-in-fat foods as a means to prevent obesity trends. ClinicoEconomics and outcomes research: CEOR. 2013;5(1):519-43.

Mansfield JL, Savaiano DA. Effect of school wellness policies and the Healthy, Hunger-Free Kids Act on food-consumption behaviors of students, 2006-2016: a systematic review. Nutrition Reviews. 2017;75(7):533-52.

Mayne SL, Auchincloss AH, Michael YL. Impact of policy and built environment changes on obesity-related outcomes: a systematic review of naturally occurring experiments. Obesity reviews: an official journal of the International Association for the Study of Obesity. 2015; 16:362-75.

Micha, R., et al. (2018). "Effectiveness of school food environment policies on children's dietary behaviors: A systematic review and meta-analysis." PLoS ONE 13(3): e0194555.

Niebylski, M. L., et al. (2014). "Healthy food procurement policies and their impact." International Journal of Environmental Research and Public Health 11(3): 2608-2627.

Niebylski, M. L., et al. (2015). "Healthy food subsidies and unhealthy food taxation: A systematic review of the evidence." Nutrition (Burbank, Los Angeles County, Calif.) 31(6): 787-795.

Osei-Assibey, G., et al. (2012). "The influence of the food environment on overweight and obesity in young children: a systematic review." BMJ Open 2(6).

Roy, R., et al. (2015). "Food Environment Interventions to Improve the Dietary Behavior of Young Adults in Tertiary Education Settings: A Systematic Literature Review." Journal of the Academy of Nutrition & Dietetics 115(10): 1647-1681.

Saraf DS, Nongkynrih B, av CS, Gupta SK, Shah B, Kapoor SK, et al. A Systematic Review of School-Based Interventions to Prevent Risk Factors Associated With Noncommunicable Diseases. Asia-Pacific Journal of Public Health. 2012;24(5):733-52.

Shemilt, I., et al. (2017). "What do we know about the effects of exposure to 'Low alcohol' and equivalent product labelling on the amounts of alcohol, food and tobacco people select and consume? A systematic review." BMC Public Health 17(1): 1-15.

Sildén KE. Impact of competitive foods in public schools on child nutrition: effects on adolescent obesity in the United States an integrative systematic literature review. Global health action. 2018;11(1):1477492.

Singh A, Bassi S, Nazar GP, Saluja K, Park M, Kinra S, et al. Impact of school policies on non-communicable disease risk factors - a systematic review. Bmc Public Health. 2017;17.

Steyn NP, Parker W, Lambert EV, McHiza Z. Nutrition interventions in the workplace: Evidence of best practice. South African Journal of Clinical Nutrition. 2009;22(3):111-7.

Tseng E, Zhang A, Shogbesan O, Gudzune KA, Wilson RF, Kharrazi H, et al. Effectiveness of Policies and Programs to Combat Adult Obesity: a Systematic Review. Journal of General Internal Medicine. 2018;33(11):1990-2001.

Verstraeten R, Roberfroid D, Lachat C, Leroy JL, Holdsworth M, Maes L, et al. Effectiveness of preventive school-based obesity interventions in low- and middle-income countries: a systematic review. The American journal of clinical nutrition. 2012;96(2):415-38.

Wright KM, Dono J, Brownbill AL, Pearson Nee Gibson O, Bowden J, Wycherley TP, et al. Sugar-sweetened beverage (SSB) consumption, correlates and interventions among Australian Aboriginal and Torres Strait Islander communities: a scoping review. BMJ open. 2019;9(2):e023630.

## Not sufficient focus on inequality (n=24)

Adam A, Jensen JD. What is the effectiveness of obesity related interventions at retail grocery stores and supermarkets? -a systematic review. BMC public health. 2016;16(1):1247.

An RP. Effectiveness of subsidies in promoting healthy food purchases and consumption: a review of field experiments. Public Health Nutrition. 2012;16(7):1215-28.

Bleich SN, Economos CD, Spiker ML, Vercammen KA, VanEpps EM, Block JP, et al. A Systematic Review of Calorie Labeling and Modified Calorie Labeling Interventions: Impact on Consumer and Restaurant Behavior. Obesity (19307381). 2017;25(12):2018-44.

Cabrera Escobar MA, Veerman JL, Tollman SM, Bertram MY, Hofman KJ. Evidence that a tax on sugar sweetened beverages reduces the obesity rate: a meta-analysis. BMC public health. 2013;13(1):1072.

Crockett RA, King SE, Marteau TM, Prevost AT, Bignardi G, Roberts NW, et al. Nutritional labelling for healthier food or non-alcoholic drink purchasing and consumption. Cochrane Database of Systematic Reviews. 2018;2:CD009315.

de Sa J, Lock K. Will European agricultural policy for school fruit and vegetables improve public health? A review of school fruit and vegetable programmes. European Journal of Public Health. 2008;18(6):558-68.

Downs SM, Bloem MZ, Zheng M, Catterall E, Thomas B, Veerman L, et al. The Impact of Policies to Reduce trans Fat Consumption: A Systematic Review of the Evidence. Current Developments in Nutrition. 2017;1(12).

Hollands GJ, Carter P, Anwer S, King SE, Jebb SA, Ogilvie D, et al. Altering the availability or proximity of food, alcohol, and tobacco products to change their selection and consumption. Cochrane Database of Systematic Reviews. 2019;9:CD012573.

Hyseni L, Bromley H, Kypridemos C, O'Flaherty M, Lloyd-Williams F, Guzman-Castillo M, et al. Systematic review of dietary trans-fat reduction interventions. Bulletin of the World Health Organization. 2017;95(12):821-30 and 30A-30G.

Mizdrak A, Scarborough P, Waterl, er WE, Rayner M. Differential Responses to Food Price Changes by Personal Characteristic: A Systematic Review of Experimental Studies. PloS one. 2015;10(7):e0130320.

Powell LM, Chriqui JF, Khan T, Wada R, Chaloupka FJ. Assessing the potential effectiveness of food and beverage taxes and subsidies for improving public health: a systematic review of prices, demand and body weight outcomes. Obesity reviews : an official journal of the International Association for the Study of Obesity. 2012;14(2):110-28

Purnell JQ, Gernes R, Stein R, Sherraden MS, Knoblock-Hahn A. A systematic review of financial incentives for dietary behavior change. Journal of the Academy of Nutrition & Dietetics. 2014;114(7):1023-35.

Redondo M, Hernández-Aguado I, Lumbreras B. The impact of the tax on sweetened beverages: a systematic review. The American journal of clinical nutrition. 2018;108(3):548-63.

Roberts KE, Ells LJ, McGowan VJ, Machaira T, Targett VC, Allen RE, et al. A rapid review examining purchasing changes resulting from fiscal measures targeted at high sugar foods and sugar-sweetened drinks. Nutrition & diabetes. 2017;7(12):302.

Sacco J, Lillico HG, Chen E, Hobin E. The influence of menu labelling on food choices among children and adolescents: a systematic review of the literature. Perspectives in Public Health. 2017;137(3):173-81.

Seyedhamzeh S, Bagheri M, Keshtkar AA, Qorbani M, Viera AJ. Physical activity equivalent labeling vs. calorie labeling: a systematic review and meta-analysis. International Journal of Behavioral Nutrition & Physical Activity. 2018;15(1):N.PAG-N.PAG.

Shemilt, I., et al. (2013). "Economic instruments for population diet and physical activity behaviour change: a systematic scoping review." PLoS ONE [Electronic Resource] 8(9): e75070.

Sisnowski J, Street JM, Merlin T. Improving food environments and tackling obesity: A realist systematic review of the policy success of regulatory interventions targeting population nutrition. PLoS ONE. 2017;12(8):1-16.

Teng AM, Jones AC, Mizdrak A, Signal L, Genç M, Wilson N. Impact of sugar-sweetened beverage taxes on purchases and dietary intake: Systematic review and meta-analysis. Obesity reviews: an official journal of the International Association for the Study of Obesity. 2019;20(9):1187-204.

von Philipsborn P, Stratil JM, Burns J, Busert LK, Pfadenhauer LM, Polus S, et al. Environmental interventions to reduce the consumption of sugar-sweetened beverages and their effects on health. The Cochrane database of systematic reviews. 2019;6:CD012292.

Waters E, de Silva-Sanigorski A, Burford BJ, Brown T, Campbell KJ, Gao Y, et al. Interventions for preventing obesity in children. Cochrane Database of Systematic Reviews. 2011;2011.

Williams AJ, Henley WE, Williams CA, Hurst AJ, Logan S, Wyatt KM. Systematic review and meta-analysis of the association between childhood overweight and obesity and primary school diet and physical activity policies. International Journal of Behavioral Nutrition and Physical Activity. 2013;10(101).

Wright A, Smith KE, Hellowell M. Policy lessons from health taxes: a systematic review of empirical studies. BMC public health. 2017;17(1):583.

## Other (n=12)

Brown, T., et al. "A systematic review of the effectiveness of interventions targeting specific out-of-home food outlets (Foodscape study)." Obesity Facts 1: 128-129. *(Could not retrieve full text)*

Ciriza, E., et al. "The challenge of promoting fruit and vegetable consumption in the school setting. A systematic review." Revista Espanola De Nutricion Comunitaria-Spanish Journal of Community Nutrition 14(1): 6-20. *(Not English language)*

Garcia, M. T. (2016). "Urban gardens and build of environments promoters of healthy eating." 178-178. *(Could not retrieve full text)*

Gittelsohn J, Rowan M, Gadhoke P. Interventions in Small Food Stores to Change the Food Environment, Improve Diet, and Reduce Risk of Chronic Disease. Preventing Chronic Disease. 2012;9. *(not possible to retrieve relevant data)*

Gittelsohn J, Trude ACB, Kim H. Pricing Strategies to Encourage Availability, Purchase, and Consumption of Healthy Foods and Beverages: A Systematic Review. Preventing chronic disease. 2017;14:E107. *(not ppossible to retrieve relevant data)*

Papoutsi GS. Food fiscal policies: A literature review. Hellenic Journal of Nutrition & Dietetics. 2012;3(2):85-91. *(Not possible to retrieve pdf)*

Rausch Herscovici, C. and I. Kovalskys (2015). "Childhood obesity. A review of school-based preventive interventions." Rev. mex. trastor. aliment 6(2): 143-151. *(not English language)*

Rodríguez Rojas, Y. L. and Y. P. Argüello Gutiérrez (2014). "Promotion and Prevention Programs for the Treatment of Childhood Obesity: a Systematic Review." Hacia promoc. salud 19(2): 111-126. *(not English language)*

Santos-Antonio, G., et al. (2019). "[Effects of front-of-package nutritional labeling of food and beverages: synopsis of systematic reviewsEfeitos da rotulagem nutricional frontal de alimentos e bebidas: sumário de estudos de revisão sistemática]." Revista panamericana de salud publica = Pan American journal of public health 43: e62. *(Not English language)*

Sebastian-Ponce, M. I., et al. (2015). "[Information perceived by consumers through food labeling on fats: a systematic review]." Nutrición hospitalaria 31(1): 129-142. *(Not English language)*

Sebastián-Ponce, M. I., et al. (2011). "[Food labeling and the prevention of overweight and obesity: a systematic review]." Cadernos de saúde pública 27(11): 2083-2094. *(Not English language)*

Wharton CM, Long M, Schwartz MB. Changing Nutrition Standards in Schools: The Emerging Impact on School Revenue. Journal of School Health. 2008;78(5):245-51. *(wrong outcome)*
