# Supplementary material for: The potential of food environment policies to reduce socioeconomic inequalities in diets and to improve healthy diets among lower socioeconomic groups: an umbrella review
Source: BMC Public Health. 2022 Mar 4;22:433. doi: 10.1186/s12889-022-12827-4 (PMC8895543; doi:10.1186/s12889-022-12827-4)
Supplement: Supplementary file 8 — Additional file 8. Study characteristics. Table describing study characteristics and key findings of included systematic reviews. [file 12889_2022_12827_MOESM8_ESM.docx]

# Additional file 8. Study characteristics

**Study characteristics and key findings of included systematic reviews**

| **Reference** | **N^o^ of relevant studies/total studies for this policy domain** | **Context**  **Search timeframe** | **Included policy interventions** | **Key findings** | **Inequality main focus of the review^1^** |
| --- | --- | --- | --- | --- | --- |
| **Food composition policy interventions** | | | | | |
| Hendry et al (2015) | 1/14 | Mainly Western, high-income countries  1980-2012 | Trans fat ban in the USA | **Across SEP^2^:**  The mean content of trans fat in consumer lunchtime fast food purchases decreased after a trans-fat ban, no difference between low and high-income neighbourhoods (neutral result)  **Low SEP:**  Not reported | N |
| McGill et al (2015) | 1/36 | Mainly Western, high-income countries  From 1980 onwards, end date not specified | Modification of food products to make them healthier (i.e. salt reformulation in the UK) | **Across SEP:**  Changes in salt intake did not differ across SEP after salt reformulation policy in the UK (neutral result)  **Low SEP:**  Not reported | Y |
| **Food labelling policy interventions** | | | | | |
| Hartmann Boyce et al (2018) | 2/35 | Mainly Western, high-income countries.  Search time frame not reported, search conducted in 2017 | Labelling/education interventions in actual or virtual/web-based grocery stores | **Across SEP:**  1 study showed negative results and 1 study showed neutral and conflicting results on food purchases, which could be due to questionable validity for the latter  **Low SEP:**  Not reported | Y |
| Olstad et al (2016) | 3/36 | Mainly Western, high-income countries.  2004-2015 | Menu labelling undertaken in three US cities  *The studies are also assessed by Sarink (2015)* | **Across SEP:**  2 “weak” and 1 “strong” quality study on menu labelling in the USA showed neutral effects on energy intake  **Low SEP:**  Not reported | Y |
| Sarink et al (2016) | 9/18 | No restriction on geography is reported, included studies are all from the USA  2003-2015 | Menu energy labelling  *Includes three studies also assessed by Olstad (2016)* | **Across SEP:**  Studies of moderate to high quality showed that menu energy labelling had none or little effect on purchases (3 studies), or reported negative or neutral results (2 studies)  **Low SEP:**  Evidence from 7 studies of moderate to high quality that menu energy labelling did not impact the energy content of purchases made by low-income customers | Y |
| **Food pricing policy interventions** | | | | | |
| Andreyeva et al 2010 | 5/160 | USA  1938-2007 | Food price changes | **FOOD TAXES**  **Across SEP:**  Review authors report that no consistent differences in price elasticities were detected due to small number of studies. Of 5 studies of unknown quality, 2 studies reported positive effects while 3 studies reported neutral effects on price elasticities across a variety of food groups  **Low SEP:**  Not reported | Y |
| Backholer et al (2016) | 8/11 | High-income countries  Database inception to June 2015 | Changes in SSB price  *Includes six studies also assessed in reviews by Olstad 2016; Thow 2014; and Eyles 2012* | **FOOD TAXES**  **Across SEP:**  Evidence from 8 observational and modelling studies of mainly medium quality that the impact of increased SSB taxes on dietary intake is neutral or positive. Mixed findings on price elasticities  **Low SEP:**  Not reported | Y |
| Black et al  (2012) | 2/14 | High-income countries  1980-2010 | Food subsidy programs in the United States | **TARGETED POLICIES**  **Across SEP:**  Not reported  **Low SEP:**  Evidence from one study (2 articles) of low quality suggests that targeted food subsidy programs is positive for fruit and vegetable consumption in low-income populations | Y |
| Cuffey et al  (2015) | 59/59 | USA  No set limit for publication date, last search conducted March 2015 | Food-related income support programs, i.e. the Supplemental Nutrition Assistance Program in the United States | **TARGETED POLICIES**  **Across SEP:**  Not reported  **Low SEP:**  Restrictions on food items that may be purchased with SNAP may have a small to moderate positive effect on household purchasing of restricted foods. Effects on diet quality and health outcomes require further investigation | Y |
| Eyles et al  (2012) | 12/32 | Member countries of the Organization for Economic Co-operation and Development (OECD)  1990-2011 | Food subsidies or food taxes, or a combination of the two  *Includes ten studies also assessed by McGill (2015); Backholer (2016); Thow (2010); Thow (2014) and Olstad (2016)* | **FOOD TAXES**  **Across SEP:**  The main body of evidence suggests that food taxes are positive in terms of inequalities (5/11 studies) or that the effect is neutral (5/11 studies); limited evidence (2/11 studies) that high SEP groups benefit more  **Low SEP:**  10 out of 12 studies reported that food pricing strategies had a positive impact on food purchase. No studies were considered as being negative for low-SEP groups, with one study being of moderately high quality | Y |
| Hartmann Boyce et al (2018) | 6/35 | Mainly Western, high-income countries.  Search time frame not reported, search conducted in 2017 | Economic interventions conducted in physical or simulated grocery stores | **FOOD SUBSIDIES**  **Across SEP:**  Limited evidence (2 studies) that the effect of price decreases was neutral  **Low SEP:**  Evidence from 4 experimental studies that subsidies had a positive impact on food purchases in low-income groups, but effects were not detected for all targeted items | Y |
| McGill et al (2015) | 7/36 | Included studies are mainly from Western, high-income countries  From 1980 onwards, end date not specified | Fiscal measures such as taxes, subsidies, or economic incentives  *Includes five studies also assessed by Eyles (2012), Thow (2010, 2014) and Backholer (2016)* | **FOOD TAXES**  **Across SEP:** 4 studies showed varying results, only significant findings reported here: 1 RCT positive effects, 2 modelling studies showed neutral effects  **Low SEP:**  Not reported  **SUBSIDIES AND TAX IN COMBINATION**  **Across SEP:** Of three modelling studies, one study showed positive and significant effects of subsidies and taxes in combination, two studies also showed positive effects but not significant  **Low SEP:**  Not reported | Y |
| Nakhimovsky et al (2016) | 6/9 | Middle-Income Countries  1990– 2016 | Taxes on Sugar-Sweetened Beverages  *Includes one study also assessed by Thow (2014)* | **FOOD TAXES**  **Across SEP:**  The majority of studies (4/6) showed positive effects in that lower socio-economic groups or more marginalized populations were more responsive to price changes in SSB products compared to higher income groups in Middle-Income Countries  **Low SEP:**  Not reported | Y |
| Olstad et al (2016) | 3/36 | Any setting or country. Included studies mainly from Western countries.  2004 to 2015 | Real-world food and beverage taxes  *Includes two studies also assessed by Backholer (2016)* | **FOOD TAXES**  **Across SEP:**  2 studies (1 weak, 1 moderate quality) found positive effects of taxes on inequalities in dietary intake and food purchases, and one study (moderate quality) found neutral effects on BMI  **Low SEP:**  Not reported | Y |
| Olstad et al (2017) | 1/18 | Developed nations  2004-2015 | Fruit and vegetables subsidies at farmer’s markets | **TARGETED POLICIES**  **Across SEP:**  No reported  **Low SEP:**  1 study of weak quality showed that a food subsidy program had no effect on F&V intake in the low-income target group | Y |
| Schultz et al (2015) | 7/18 | United States  2009-2014 | Special Supplemental Nutrition Program for Women, Infants, and  Children (WIC) Food Package Revision | **TARGETED POLICIES**  **Across SEP:**  No reported  **Low SEP:**  Evidence from 7 studies (unknown quality) that revisions in the WIC scheme led to positive changes in food purchases and in food consumption among the target group, however improvements were minimal | Y |
| Thow et al (2010) | 5/20 | No limitations, majority of included studies set in Western, high-income countries, over half in United States  2003-2013 | Food taxes and subsidies  *Includes four studies also assessed by Eyles (2012) and McGill (2015))* | **FOOD TAXES**  **Across SEP:**  Limited evidence (no quality assessment) from 2 studies that food taxes are economically regressive  **Low SEP:**  Not reported  **SUBSIDIES AND TAX IN COMBINATION**  **Across SEP:**  Limited evidence (little detail in review) from 3 modelling studies that taxes and subsidies in combination are positive in terms of inequality  **Low SEP:**  Not reported | N |
| Thow et al (2014) | 11/43 | No limitations, included studies are set in Europe and USA.  2009-2012 | Food taxes and subsidies  *Includes seven studies also assessed by Backholer (2016), Nakhimovsky (2016), Eyles (2012) and McGill (2015).* | **FOOD TAXES**  **Across SEP:**  Of 10 modelling studies 9 are reported to show positive effects on inequalities in food purchases  **Low SEP:**  Not reported  **FOOD SUBSIDIES**  **Across SEP:**  2 modelling studies and 1 “stated preference” study suggest that universal subsidies are negative but that targeted subsidies is positive in terms of inequalities | N |
| **Food provision policy interventions** | | | | | |
| Olstad et al (2016) | 11/36 | Any setting or country. Included studies are from a range of countries, mainly Western.  2004 to 2015 | School nutrition interventions or policies including free fruit schemes | **Across SEP:**  The majority of studies (6 of 11, weak to moderate quality) reported neutral results, mainly on dietary outcomes. 4 studies (2 “weak”, 2 “strong” quality) reported negative effects on dietary outcomes  **Low SEP:**  Not reported | Y |
| Olstad et al (2017) | 9/18 | Developed countries  2004-2015 | Organizational policies in schools, often implemented within multicomponent interventions | **Across SEP:**  Not reported  **Low SEP:**  Three studies of strong quality assessed free fruit and vegetable schemes and found overall positive results on diet-related outcomes. Six studies on diverse school food policies within multicomponent interventions, mainly of strong or moderate quality, found mainly positive impacts on diet-related or anthropometric outcomes, with some interventions showing no impact | Y |
| **Food retail policy interventions** | | | | | |
| Abeykoon et al (2017) | 10/11 | Low-income neighbourhoods in England, Scotland, USA.  1995-2015 | Opening of new grocery stores or interventions in existing grocery stores in low-income neighbourhoods  *Includes two studies also assessed by Olstad et al (2017).* | **Across SEP:**  Not reported  **Low SEP:**  Five studies of mixed quality showed some positive diet changes for participants in low-income neighbourhoods; however, most studies were NS or had small effect sizes, six studies no change or negative outcome | Y^3^ |
| Hartmann-Boyce et al (2018) | 3/35 | Mainly Western, high-income countries  Search time frame not reported | Store environment changes in grocery stores | **Across SEP:**  Not reported  **Low SEP:**  Of the 3 studies, all with high risk of bias, 1 found small, significant effects on food purchases but the other studies detected no effect | Y |
| Olstad et al (2017) | 2/18 | Developed countries  2004-2015 | Financial support to the retail sector to open new supermarkets in disadvantaged neighbourhoods  *Includes two studies also assessed by Abeykoon et al (2017)* | **Across SEP:**  Not reported  **Low SEP:**  One study found no effect, another study of moderate quality found limited positive effect on dietary outcomes | Y |

Notes:

Each review may cover different policy domains, see Table 2 in main article.

^1^ Y= Yes. Inequality is either the main objective or a subobjective of the study. N= No, inequality only considered in results or discussion but not considered in objectives or methods.

^2^ SEP = socioeconomic position

Positive/⇧: The effect of policy intervention larger in lower vs. higher SEP groups; reducing socioeconomic inequalities in diets;

Neutral/⬄: No difference in effects in outcomes across SEP

Negative/⇩: The effect of the policy intervention higher in higher vs. lower SEP groups; increasing socioeconomic inequalities in diets.

Inconclusive/~: The results were inconsistent so that no overall conclusion could be estimated

No effect/0: No effect of policy intervention detected.

^3^Inequality is an overall theme of this review and all primary studies are set in low-income neighbourhoods, however inequality is not integrated in research question, inclusion criteria or methods.
