# Supplementary material for: The potential of food environment policies to reduce socioeconomic inequalities in diets and to improve healthy diets among lower socioeconomic groups: an umbrella review
Source: BMC Public Health. 2022 Mar 4;22:433. doi: 10.1186/s12889-022-12827-4 (PMC8895543; doi:10.1186/s12889-022-12827-4)
Supplement: Supplementary file 9 — Additional file 9. List of primary studies reported in reviews. A list showing all primary studies that form the evidence base of the included systematic reviews included in the umbrella review, also showing what primary studies have been reported in more than one review. [file 12889_2022_12827_MOESM9_ESM.docx]

# Additional file 9. List of primary studies reported in reviews

Only primary studies that are relevant for the umbrella review have been listed

**Abeykoon et al (2017)**

| **Primary studies as referenced in review** | **Primary study also reported by** |
| --- | --- |
| **Food in retail** | |
| 36: Elbel B, Moran A, Dixon LB et al. (2015) Assessment of a government-subsidized supermarket in a high-need area on household food availability and children’s dietary intakes. Public Health Nutr18, 2881–2890 | Olstad et al (2017) |
| 37: Dubowitz T, Ghosh-Dastidar M, Cohen DAet al. (2015) Diet and perceptions change with supermarket introduction in a food desert, but not because of supermarket use. Health Aff (Millwood)34, 1858–1868. |  |
| 30: Cummins S, Flint E & Matthews SA (2014) New neighborhood grocery store increased awareness of food access but did not alter dietary habits or obesity. Health Aff (Millwood)33, 283–291 | Olstad et al (2017) |
| 31: Cummins S, Petticrew M, Higgins Cet al. (2005) Largescale food retailing as an intervention for diet and health: quasi-experimental evaluation of a natural experiment. J Epidemiol Community Health59, 1035–1040 |  |
| 38: Cummins S, Findlay A, Higgins C et al. (2008) Reducing inequalities in health and diet: findings from a study on the impact of a food retail development. Environ Plann A40,402–422 |  |
| 34: Sadler RC, Gilliland JA & Arku G (2013) A food retail-based intervention on food security and consumption. Int J Environ Res Public Health10, 3325–3346. |  |
| 33. Wang MC, MacLeod KE, Steadman Cet al. (2007) Is the opening of a neighborhood full-service grocery store followed by a change in the food behavior of residents? J Hunger Environ Nutr2,3–18. |  |
| 32. Wrigley N, Warm D & Margetts B (2003) Deprivation, diet, and food-retail access: findings from the Leeds ‘food deserts’ study. Environ Plann A 35, 151–188. |  |
| 35. Wrigley N, Warm D, Margetts B et al. (2002) Assessing the impact of improved retail access on diet in a ‘food desert’: a preliminary report. Urban Stud 39, 2061–2082. |  |
| 29. Gill L & Rudkin S (2014) Deconstructing supermarket intervention effects on fruit and vegetable consumption in areas of limited retail access: evidence from the Seacroft Study. Environ Plann A 46, 649–665. |  |

**Andreyeva et al 2010**

| **Primary studies as referenced in review** | **Primary study also reported by** |
| --- | --- |
| **Food prices** | |
| 18. Huang KS, Lin B. Estimation of Food Demand and Nutrient Elasticities From Household Survey Data. Washington, DC: US Dept of Agriculture; 2000. |  |
| 20. Park JL, Holcomb RB, Raper KC, Capps O Jr. A demand systems analysis of food commodities by US households segmented by income. Am JAgric Econ. 1996;78(2):290–300. |  |
| 34. Bartlett RW. Fluid milk sales related to demand elasticities. JDairy Sci. 1965; 47(12):1314–1321 |  |
| 35. Barnes R, Gillingham R. Demographic effects in demand analysis: estimation of the quadratic expenditure system using microdata. RevEcon Stat. 1984; 66(4):591–601. |  |
| 36. Raper KC, Wanzala MN, Nayga RM Jr. Food expenditures and household demographic composition in the US: a demand systems approach. Appl Econ. 2002;34(8):981–992. |  |

**Backholer et al (2016)**

| **Primary studies as referenced in review** | **Primary study also reported by** |
| --- | --- |
| **Food prices** | |
| 22. Lin BH, Smith TA, Lee JY et al. (2011) Measuring weight outcomes for obesity intervention strategies: the case of a sugar-sweetened beverage tax. Econ Hum Biol9, 329–341. | Thow et al (2014) |
| 28: Sturm R, Powell LM, Chriqui JF et al. (2010) Soda taxes, soft drink consumption, and children’s body mass index. HealthAff (Millwood)29, 1052–1058. | Thow et al (2014)  Olstad et al (2016) |
| 30. Ni Mhurchu C, Eyles H, Schilling C et al. (2013) Food prices and consumer demand: differences across income levels and ethnic groups. PLoS One 8, e75934. |  |
| 31. Finkelstein EA, Zhen C, Nonnemaker Jet al. (2010). Impact of targeted beverage taxes on higher- and lower-income households. Arch Intern Med170, 2028–2034. | Eyles et al (2012)  Thow et al (2014)  McGill et al (2015) |
| 32. Zhen C, Wohlgenant M, Karns Set al. (2011) Habit formation and demand for sugar-sweetened beverages. Am JAgric Econ93, 175–193. | Eyles et al (2012) |
| 33. Briggs AD, Mytton OT, Kehlbacher A et al. (2013) Overall and income specific effect on prevalence of overweight and obesity of 20% sugar sweetened drink tax in UK: econometric and comparative risk assessment modelling study. BMJ347, f6189. |  |
| 34. Briggs AD, Mytton OT, Madden Det al. (2013) The potential impact on obesity of a 10% tax on sugar-sweetened beverages in Ireland, an effect assessment modelling study. BMC Public Health13, 860. |  |
| 36. Zhen C, Finkelstein EA, Nonnemaker Jet al. (2014) Predicting the effects of sugar-sweetened beverage taxes on food and beverage demand in a large demand system. Am JAgric Econ96, 1–25. |  |

**Black et al (2012)**

| **Primary studies as referenced in review** | **Primary study also reported by** |
| --- | --- |
| **Food prices** | |
| 32. Herman DR, Harrison GG, Afifi AA, Jenks E: Effect of a targeted subsidy on intake of fruits and vegetables among low-income women in the special supplemental nutrition program for women, infants, and children. Am JPublic Health2008,98(1):98–105. |  |
| 40. Herman DR, Harrison GG, Jenks E: Choices made by Low-income women provided with an economic supplement for fresh fruit and vegetable purchase. J Am Diet Assoc2006,106(5):740–744. |  |

Cuffey et al (2015)

The references in this study were only visually inspected, because these studies are thematically different from studies in other reviews. The inspection identified no overlapping studies.

**Eyles et al (2012)**

| **Primary studies as referenced in review** | **Primary study also reported by** |
| --- | --- |
| **Food prices** | |
| 29. Allais O, Bertail P, Nichele V (2010) The effects of a fat tax on French households’ purchases: a nutritional approach. Am J Agric Econ 92: 228–244. | McGill et al (2015) |
| 31. Chouinard HH, Davis DE, LaFrance JT, Perloff JM (2007) Fat taxes: big money for small change. Forum Health Econ Policy 10: article 2. | Thow et al (2010) |
| 47. Dong D, Lin B-H (2009) Fruit and vegetable consumption by low-income Americans. Washington (District of Columbia): US Department of Agriculture. | Thow et al (2010) |
| 13. Fantuzzi K (2008) Carbonated soft drink consumption: implications for obesity policy [PhD dissertation]. Storrs (Connecticut): University of Connecticut |  |
| 38. Finkelstein EA, Zhen C, Nonnemaker J, Todd JE (2010) Impact of targeted beverage taxes on higher- and lower-income households. Arch Intern Med 170:2028–2034. | Backholer et al (2016)  McGill et al (2015)  Thow et al (2014) |
| 51. LaCroix A, Muller L, Ruffieux B (2010) To what extent would the poorest consumers nutritionally and socially benefit from a global food tax and subsidy reform? A framed field experiment based on daily food intake. Available: http://ideas.repec.org/p/gbl/wpaper/201004.html. Accessed 25 January 2012. | Thow et al (2014) |
| 40. Nnoaham KE, Sacks G, Rayner M, Mytton O, Gray A (2009) Modelling income group differences in the health and economic impacts of targeted food taxes and subsidies. Int J Epidemiol 38: 1324–1333. | McGill et al (2015)  Thow et al (2010) |
| 48. Nordstrom J, Thunstrom L (2010) Can targeted food taxes and subsidies improve the diet? Distributional effects among income groups. Food Policy 36:259–271. |  |
| 49. Sassi F, Cecchini M, Lauer J, Chisholm D (2009) Improving lifestyles, tackling obesity: the health and economic impact of prevention strategies. OECD Health Working Papers No. 48. doi:10.1787/220087432153 |  |
| 42. Smed S, Jensen J, Denver S (2007) Socio-economic characteristics and the effect of taxation as a healthy policy instrument. Food Policy 32: 624–639. | McGill et al (2015)  Thow et al (2010) |
| 58. Tefft N (2008) The effects of a soft drink tax on household expenditures. Lewiston (Maine): Bates College. Available: http://abacus.bates.edu/,ntefft/research/soft_drink_taxes_ces.pdf. Accessed 28 January 2012. |  |
| 44. Zhen C, Wohlgenant MK, Karns S, Kaufman P (2011) Habit formation and demand for sugar-sweetened beverages. Am J Agric Econ 93: 175–193. | Backholer et al (2016) |

**Hartmann-Boyce (2018)**

| **Primary studies as referenced in review** | **Primary study also reported by** |
| --- | --- |
| **Food prices** | |
| 19. Anderson ES, Winett RA, Bickley PG, Walberg-Rankin J, Moore JF, Leahy M, Harris CE, Gerkin RE. The effects of a multimedia system in supermarkets to alter shoppers’ food purchases: nutritional outcomes and caveats. J Health Psychol 1997; 2:209–23. |  |
| 23. Budd N, Jeffries JK, Jones-Smith J, Kharmats A, McDermott AY, Gittelsohn J. Store-directed price promotions and communications strategies improve healthier food supply and demand: impact results from a randomized controlled, Baltimore City store-intervention trial. Public Health Nutr 2017:1–11. |  |
| 37. Ni Mhurchu C, Blakely T, Jiang Y, Eyles HC, Rodgers A. Effects of price discounts and tailored nutrition education on supermarket purchases: a randomized controlled trial. Am J Clin Nutr 2010;91:736–  47. |  |
| 44. Waterlander WE, Steenhuis IHM, de Boer MR, Schuit AJ, Seidell JC. The effects of a 25% discount on fruits and vegetables: results of a randomized trial in a three-dimensional web-based supermarket. Int JBehav Nutr Phys Act 2012;9:11. |  |
| 45. Waterlander WE, Steenhuis IHM, de Boer MR, Schuit AJ, Seidell JC. Introducing taxes, subsidies or both: the effects of various food pricing strategies in a web-based supermarket randomized trial. Prev Med 2012;54:323–30. |  |
| 47. Waterlander WE, de Boer MR, Schuit AJ, Seidell JC, Steenhuis IHM. Price discounts significantly enhance fruit and vegetable purchases when combined with nutrition education: a randomized controlled supermarket trial. Am J Clin Nutr 2013;97:886–95. |  |
| Labelling | |
| 16. Ducrot P, Julia C, Mejean C, Kesse-Guyot E, Touvier M, Fezeu LK,Hercberg S, Péneau S. Impact of different front-of-pack nutrition labels on consumer purchasing intentions: a randomized controlled trial. AmJ Prev Med 2016;50:627–36. |  |
| 38. Ni Mhurchu CN, Ekaterina V, Yannan J et al. Effects of interpretive nutrition labels on consumer food purchases: the Starlight randomized controlled trial. Am J Clin Nutr 2017;105:695–704. |  |
| Food Retail | |
| 23. Budd N, Jeffries JK, Jones-Smith J, Kharmats A, McDermott AY, Gittelsohn J. Store-directed price promotions and communications strategies improve healthier food supply and demand: impact results from a randomized controlled, Baltimore City store-intervention trial. Public Health Nutr 2017:1–11. |  |
| 33. Lent MR, Vander Veur SS, McCoy TA, Wojtanowski AC, Sandoval B, Sherman S, Komaroff E, Foster GD. A randomized controlled study of a healthy corner store initiative on the purchases of urban, low-income youth. Obesity 2014;22:2494–500. |  |
| 42. Thorndike AN, Bright O-JM, Dimond MA, Fishman R, Levy DE. Choice architecture to promote fruit and vegetable purchases by families participating in the Special Supplemental Program for Women, Infants, and Children (WIC): randomized corner store pilot study. Public Health Nutr 2017;20:1297–305. |  |

**Hendry et al (2015)**

| **Primary studies as referenced in review** | **Primary study also reported by** |
| --- | --- |
| **Food composition** | |
| 25. Angell SY, Cobb LK, Curtis CJ, KontyKJ, Silver LD. Change in trans fatty acid content of fast-food purchases associated with New York City’s restaurant regulation: a pre---post study. Ann Intern Med.2012;157(2):81---86 |  |

**McGill et al (2015)**

| **Primary studies as referenced in review** | **Primary study also reported by** |
| --- | --- |
| **Food prices** | |
| 34. Allais O, Bertail P, Nichèle V. The effects of a fat tax on french households’ purchases: a nutritional approach. Am J Agric Econ. 2010;92:228–45. | Eyles et al (2012) |
| 35. Dallongeville J, Dauchet L, Mouzon O, Réquillart V, Soler L-G. Increasing fruit and vegetable consumption: a cost-effectiveness analysis of public policies. Eur J Pub Health. 2011;21:69–73. | Thow et al (2014) |
| 36. Nederkoorn C, Havermans RC, Giesen JCAH, Jansen A. High tax on high energy dense foods and its effects on the purchase of calories in a supermarket. An experiment Appetite. 2011;56:760–5.’ |  |
| 37. Nnoaham KE, Sacks G, Rayner M, Mytton O, Gray A. Modelling income group differences in the health and economic impacts of targeted food taxes and subsidies. Int J Epidemiol. 2009;38:1324–33 | Eyles et al (2012)  Thow et al (2014) |
| 38. Smed S, Jensen JD, Denver S. Socio-economic characteristics and the effect of taxation as a health policy instrument. Food Policy. 2007;32:624–39. | Eyles et al (2012)  Thow et al (2010) |
| 39. Tiffin R, Salois M. Inequalities in diet and nutrition. Proc Nutr Soc.2012;71:105–11. |  |
| 41. Finkelstein Ea ZC. Impact of targeted beverage taxes on higher- and lower-income households. Arch Intern Med. 2010;170:2028–34. | Backholer et al (2016)  Eyles (2012)  Thow et al (2014) |
| **Food composition** | |
| 49. Millett C, Laverty AA, Stylianou N, Bibbins-Domingo K, Pape UJ. Impacts of a national strategy to reduce population salt intake in England: serial cross-sectional study. PLoS ONE. 7: e29836. |  |

**Nakhimovsky (2016)**

| **Primary studies as referenced in review** | **Primary study also reported by** |
| --- | --- |
| **Food prices** | |
| 35.Colchero MA, Popkin BM ,Rivera JA,Ng. Beverage purchases from stores in Mexico under the excise tax on sugar sweetened beverages: observational study. BMJ. 2016;352:h6704. doi:10.1136/bmj.h6704PMID:26738745. |  |
| 36. Barquera S, Hernandez-Barrera L, Tolentino ML, Espinosa J, Ng SW, Rivera JA, et al. Energy Intake from Beverages Is Increasing among Mexican Adolescents and Adults. Journal of Nutrition. 2008;138(12):2454–61.doi:10.3945/jn.108.092163PMID:19022972 |  |
| 39. Claro RM, Levy RB, Popkin BM, Monteiro CA. Sugar-Sweetened Beverage Taxes in Brazil. American Journal of PublicHealth.2012;102(1):178–83.doi:10.2105/ajph.2011.300313 PMID:22095333 | Thow et al (2014) |
| 40.Basu S, Vellakkal S, Agrawal S, Stuckler D, Popkin B, EbrahimS. Averting Obesity and Type2 Diabetes in India through Sugar-Sweetened Beverage Taxation: An Economic-Epidemiologic Modeling Study. PLoSMedicine. 2014;11(1).e100158210.1371/journal.pmed.1001582.PMID:WOS:000337975600002. |  |
| 44. Paraje G. The Effect of Price and Socio-Economic Level on the Consumption of Sugar-Sweetened Beverages (SSB): The Case of Ecuador.PLoSOne.2016;11(3):e0152260.Epub2016/03/31.doi:10.1371/journal.pone.0152260PMID:27028608. |  |
| 45.Colchero MA, Salgado JC, Unar-Munguı ́a M, Herna ́ndez-A ́vila M, Rivera-Dommarco JA. Price elasticity of the demand for sugar sweetened beverages and soft drinks in Mexico. Economics& HumanBiol-ogy.2015;19:129–37.doi:10.1016/j.ehb.2015.08.007 |  |

**Olstad et al (2016)**

| **Primary studies as referenced in review** | **Primary study also reported by** |
| --- | --- |
| **Food labelling** | |
| 72. Dumanovsky T, Huang CY, Nonas CA, Matte TD, BassettMT, Silver LD. Changes in energy content of lunchtime purchases from fast food restaurants after introduction of calorie labelling: cross sectional customer surveys.BMJ2011;343:d4502–5367. | Sarink et al (2015) |
| 73. Elbel B, Mijanovich T, Dixon L B et al. Calorie labeling, fast food purchasing and restaurant visits. Obesity 2013; 21: 2172–9. | Sarink et al (2015) |
| 74. Krieger JW, Chan NL, Saelens BE, Ta ML, Solet D, Fleming DW. Menu labeling regulations and calories purchased at chain res-taurants. Am J Prev Med2013;44: 595–604. | Sarink et al (2015) |
| **Food prices** | |
| 65. Bíró A. Did the junk food tax make the Hungarians eat healthier? Food Policy2015;54: 107–15. |  |
| 66. Sturm R, Powell LM, Chriqui JF, Chaloupka FJ. Soda taxes, soft drink consumption, and children’s body mass index. HealthAff (Millwood)2010;29: 1052–8. | Backholer et al (2016)  Thow et al (2014) |
| **Food provision** | |
| 60. Bae SG, Kim JY, Kim KY, Park SW, Bae J, Lee WK. Changes in dietary behavior among adolescents and their association with government nutrition policies in Korea, 2005–2009.J Prev Med PubHealth2012;45:47–59. |  |
| 82. Bere E, Hilsen M, Klepp KI. Effect of the nationwide free school fruit scheme in Norway. Br J Nutr2010;104: 589–94. |  |
| 69. Cullen KW, Watson KB, Fithian AR. The impact of school socioeconomic status on student lunch consumption after implementation of the Texas Public School Nutrition Policy. J SchHealth2009;79: 525–31 quiz 61-3. |  |
| 45. Fogarty AW, Antoniak M, Venn AJ et al. Does participation in a population-based dietary intervention scheme have a lasting impact on fruit intake in young children? Int J Epidemiol2007;36: 1080–5. |  |
| 83. Hughes RJ, Edwards KL, Clarke GP, Evans CE, Cade JE, Ransley JK. Childhood consumption of fruit and vegetables across England: a study of 2306 6-7-year-olds in 2007.Br J Nutr2012;108: 733–42. | McGill et al (2015) |
| 58. Kim K, Park SM, Oh KW. The impact of nutritional policy on socioeconomic disparity in the unhealthy food intake among Korean adolescents. Appetite2013;71: 388–95. |  |
| 68. Mendoza JA, Watson K, Cullen KW. Change in dietary energy density after implementation of the Texas Public School Nutrition Policy.J Am Diet Assoc2010;110: 434–40. |  |
| 59. Schagen S, Blenkinsop S, Schagen Iet al. Evaluating the impact of the National Healthy School Standard: using national datasets. Health Educ Res2005;20: 688–96. |  |
| 79. Taber DR, Chriqui JF, Powell L, Chaloupka FJ. Association between state laws governing school meal nutrition content and student weight status: implications for new USDA school meal standards. JAMA Pediatr2013;167: 513–9. |  |

**Olstad et al (2017)**

| **Primary studies as referenced in review** | **Primary study also reported by** |
| --- | --- |
| **Food prices** | |
| 49. Olsho LE, Payne GH, Walker DK, Baronberg S, Jernigan J, Abrami A. Impacts of a farmers’ market incentive programme on fruit and vegetable access, purchase and consumption. PublicHealth Nutr2015;18: 2712–2721. |  |
| **Food in retail** | |
| 50. Cummins S, Flint E, Matthews SA. New neighborhood grocery store increased awareness of food access but did not alter dietary habits or obesity. Health Aff 2014;33: 283–291. | Abeykoon et al (2017) |
| 51. Elbel B, Moran A, Dixon LBet al. Assessment of a government-subsidized supermarket in a high-need area on household food availability and children’s dietary intakes. PublicHealth Nutr2015;18: 2881–2890. | Abeykoon et al (2017) |
| **Food provision** | |
| 34. Wright K, Giger JN, Norris K, Suro Z. Impact of a nurse-directed, coordinated school health program to enhance physical activity behaviors and reduce body mass index among minority children: a parallel-group, randomized control trial. Int J Nurs Stud2013;50: 727–737. |  |
| 35. Wright K, Norris K, Newman Giger J, Suro Z. Improving healthy dietary behaviors, nutrition knowledge, and self-efficacy among underserved school children with parent and community involvement.Child2012;8: 347–356. |  |
| 36. Wright K, Suro Z. Using community–academic partnerships and a comprehensive school-based program to decrease health disparities in activity in school-aged children. J Prev IntervCommunity2014;42: 125–139. |  |
| 37. Alaimo K, Carlson JJ, Pfeiffer KAet al. Project FIT: a school, community and social marketing intervention improves healthy eating among low-income elementary school children. JCommunity Health2015;40: 815–826. |  |
| 38. Alaimo K, Oleksyk SC, Drzal NBet al. Effects of changes in lunch-time competitive foods, nutrition practices, and nutrition policies on low-income middle-school children’s diets.Child2013;9: 509–523. |  |
| 40. Foster GD, Sherman S, Borradaile KE et al.A policy-based school intervention to prevent overweight and obesity.Pediatrics.2008;121: e794–e802. |  |
| 47. Olsho LE, Klerman JA, Ritchie L, Wakimoto P, Webb KL, Bartlett S. Increasing child fruit and vegetable intake: findings from the US Department of Agriculture Fresh Fruit and Vegetable Program. J Acad Nutr Diet2015;115: 1283–1290. |  |
| 48. Ashfield-Watt PA, Stewart EA, Scheffer JA. A pilot study of the effect of providing daily free fruit to primary-school children in Auckland, New Zealand. Public Health Nutr2009;12: 693–701. |  |

**Sarink et al (2016)**

| **Primary studies as referenced in review** | **Primary study also reported by** |
| --- | --- |
| **Food labelling** | |
| Vadiveloo, M. K., Dixon, L. B., & Elbel, B. (2011). Consumer purchasing patterns in response to calorie labeling legislation in New York City. International Journal of Behavioral Nutrition and Physical Activity, 8, 51. doi:1479-5868-8-51 [pii]10.1186/1479-5868-8-51 [doi]. |  |
| Dumanovsky, T., Huang, C. Y., Nonas, C. A., Matte, T. D., Bassett, M. T., & Silver, L. D.(2011). Changes in energy content of lunchtime purchases from fast food restaurants after introduction of calorie labelling: cross sectional customer surveys. BMJ, 343, d4464. | Olstad et al (2016) |
| Elbel, B. (2011). Consumer estimation of recommended and actual calories at fast food restaurants. Obesity (Silver Spring), 19(10), 1971e1978. doi:oby2011214 [pii]10. 1038/oby.2011.214 [doi]. |  |
| Elbel, B., Gyamfi, J., & Kersh, R. (2011). Child and adolescent fast-food choice and the influence of calorie labeling: a natural experiment. International Journal of Obesity (London), 35(4), 493e500. doi:ijo20114 [pii]10. 1038/ijo.2011.4 [doi]. |  |
| Elbel, B., Kersh, R., Brescoll, V. L., & Dixon, L. B. (2009). Calorie labeling and food choices: a first look at the effects on low-income people in New York City. Health Affairs (Millwood), 28(6), w1110e1121. doi:hlthaff.28.6.w1110 [pii]10.1377/hlthaff.28.6.w1110 [doi]. |  |
| Elbel, B., Mijanovich, T., Dixon, L. B., Abrams, C., Weitzman, B.,Kersh, R....Ogedegbe, G. (2013). Calorie labeling, fast food purchasing and restaurant visits. Obesity (Silver Spring),2172e2179.http://dx.doi.org/10.1002/oby.20550. | Olstad et al (2016) |
| Krieger, J. W., Chan, N. L., Saelens, B. E., Ta, M. L., Solet, D., & Fleming, D. W. (2013).Menu labeling regulations and calories purchased at chain restaurants. American Journal of Preventive Medicine, 44(6), 595e604.http://dx.doi.org/10.1016/j.amepre.2013.01.031 | Olstad et al (2016) |
| Bollinger, B., Leslie, P., & Sorensen, A. (2011). Calorie posting in chain restaurants. American Economical Journal Economic Policy, 3(1), 91e128.http://dx.doi.org/10.1257/pol.3.1.91. |  |
| Harnack, L. J., French, S. A., Michael, J. M., Story, M. T., Jeffery, R. W., & Rydell, S. A.(2008). Effects of calorie labeling and value size pricing on fast food meal choices: results from an experimental trial. International Journal of Behavioral Nutrition and Physical Activity, 5. |  |

**Schultz et al (2015)**

| **Primary studies as referenced in review** | **Primary study also reported by** |
| --- | --- |
| **Food prices** | |
| 7. Andreyeva T, Luedicke J. Federal food package revisions effects on purchases of whole-grain products. Am J Prev Med. 2013;45(4):422-429. |  |
| 8. Andreyeva T, Luedicke J, Tripp AS, Henderson KE. Effects of reduced juice allowances in food packages for the Women, Infants, and Children program. Pediatrics. 2013;131(5):919-927. |  |
| 9. Ishdorj A, Capps O. The effect of revised WIC food packages on Native American children. Am J Agric Econ. 2013;95(5):1266-1272 |  |
| 10. Odoms-Young AM, Kong A, Schiffer LA, et al. Evaluating the initial impact of the revised Special Supplemental Nutrition Program for Women, Infants, and Children (WIC) food packages on dietary intake and home food availability in African-American and Hispanic families. Public Health Nutr. 2014;17(01):83-93. |  |
| 11. Andreyeva T, Luedicke J, Henderson KE, Schwartz MB. The positive effects of the revised milk and cheese allowances in the Special Supplemental Nutrition Program for Women, Infants, and Children. J Acad Nutr Diet. 2014;114(4):622-630. |  |
| 12. Whaley SE, Ritchie LD, Spector P, Gomez J. Revised WIC food package improves diets of WIC families. J Nutr Educ Behav. 2012;44(3):204-209. |  |
| 13. Chiasson MA, Findley SE, Sekhobo JP, et al. Changing WIC changes what children eat. Obesity. 2013;21(7):1423-1429. |  |

**Thow et al 2010**

| **Primary studies as referenced in review** | **Primary study also reported by** |
| --- | --- |
| **Food prices** | |
| 20. Nnoaham KE, Sacks G, Rayner M, Mytton O, Gray A. Modelling income group differences in the health and economic impacts of targeted food taxes and subsidies. Int J Epidemiol 2009;38:1324–33. doi:10.1093/ije/dyp214 PMID:19483200 | Eyles et al (2012)  McGill et al (2015) |
| 24. Smed S, Jensen JD, Denver S. Socio-economic characteristics and the effect of taxation as a health policy instrument. Food Policy 2007;32:624–39. doi:10.1016/j.foodpol.2007.03.002 | Eyles et al (2012)  McGill et al (2015) |
| 25. Chouinard HH, Davis DE, LaFrance JT, Perloff JM. Fat taxes: big money for small change. Forum Health Econ Policy 2007;10:2. | Eyles et al (2012) |
| 33. Dong D, Lin B. Fruit and vegetable consumption by low-income Americans: would a price reduction make a difference? Washington: United States Department of Agriculture Economic Research Service; 2009. | Eyles et al (2012) |
| 40. Farra J, Jones J, Mishra A. An analysis of policy options for reducing obesity in Georgia. 2005 (Unpublished report, accessed initially through Google Scholar). |  |

**Thow et al (2014)**

| **Primary studies as referenced in review** | **Primary study also reported by** |
| --- | --- |
| **Food prices** | |
| 26. Lin BH, Smith TA, Lee JY, et al. Measuring weight outcomes for obesity intervention strategies: the case of a sugar-sweetened beverage tax. Econ Human Biol.2011;9:329–341 | Backholer et al (2016) |
| 20. Claro RM, Levy RB, Popkin BM, et al. Sugar-sweetened beverage taxes in Brazil. Am J Public Health. 2012;102:178–183. | Nakhimovsky et al (2016) |
| 25. Kotakorpi K, Härkänen T, Pietinen P, et al. The welfare effects of health-based food tax policy. CESifo Working Paper No. 3633. Paper presented at: CESifo Area Conference on Public Sector Economics; April 8–10, 2011; Munich; 2011. |  |
| 41. Khan R, Misra K, Singh V. Will a Fat Tax Work? Paper presented at: Summer Institute in Competitive Strategy 2012; July 9–13, 2012; Berkeley, CA. |  |
| 48. Sturm R, Powell LM, Chriqui JF, et al. Soda taxes, soft drink consumption, and children’s body mass index. Health Aff. 2010;29:1052–1058. | Backholer et al (2016) Olstad et al (2016) |
| 23. Finkelstein EA, Zhen C, Nonnemaker J, et al. Impact of targeted beverage taxes on higher- and lower-income households. Arch Intern Med. 2010;170:2028–2034. | Backholer et al (2016)  Eyles et al (2012)  McGill et al (2015) |
| 29. Nordström J, Thunström L. Can targeted food taxes and subsidies improve the diet? Distributional effects among income groups. Food Policy. 2011;36:259–271. |  |
| 32. Dallongeville J, Dauchet L, de Mouzon O, et al. Increasing fruit and vegetable consumption: a cost-effectiveness analysis of public policies. Eur J Public Health.2011;21:69–73. | McGill et al (2015) |
| 50. Lacroix A, Muller L, Ruffieux B. To what extent would the poorest consumers nutritionally and socially benefit from a global food tax and subsidy reform? A framed field experiment based on daily food intake. Association Francaised’Economie Expérimentale Research Paper no. 2010-05. Grenoble: GrenobleApplied Economics Laboratory (GAEL); 2010. | Eyles et al (2012) |

# Additional file 9. List of primary studies reported in reviews

Only primary studies that are relevant for the umbrella review have been listed

**Abeykoon et al (2017)**

| **Primary studies as referenced in review** | **Primary study also reported by** |
| --- | --- |
| **Food in retail** | |
| 36: Elbel B, Moran A, Dixon LB et al. (2015) Assessment of a government-subsidized supermarket in a high-need area on household food availability and children’s dietary intakes. Public Health Nutr18, 2881–2890 | Olstad et al (2017) |
| 37: Dubowitz T, Ghosh-Dastidar M, Cohen DAet al. (2015) Diet and perceptions change with supermarket introduction in a food desert, but not because of supermarket use. Health Aff (Millwood)34, 1858–1868. |  |
| 30: Cummins S, Flint E & Matthews SA (2014) New neighborhood grocery store increased awareness of food access but did not alter dietary habits or obesity. Health Aff (Millwood)33, 283–291 | Olstad et al (2017) |
| 31: Cummins S, Petticrew M, Higgins Cet al. (2005) Largescale food retailing as an intervention for diet and health: quasi-experimental evaluation of a natural experiment. J Epidemiol Community Health59, 1035–1040 |  |
| 38: Cummins S, Findlay A, Higgins C et al. (2008) Reducing inequalities in health and diet: findings from a study on the impact of a food retail development. Environ Plann A40,402–422 |  |
| 34: Sadler RC, Gilliland JA & Arku G (2013) A food retail-based intervention on food security and consumption. Int J Environ Res Public Health10, 3325–3346. |  |
| 33. Wang MC, MacLeod KE, Steadman Cet al. (2007) Is the opening of a neighborhood full-service grocery store followed by a change in the food behavior of residents? J Hunger Environ Nutr2,3–18. |  |
| 32. Wrigley N, Warm D & Margetts B (2003) Deprivation, diet, and food-retail access: findings from the Leeds ‘food deserts’ study. Environ Plann A 35, 151–188. |  |
| 35. Wrigley N, Warm D, Margetts B et al. (2002) Assessing the impact of improved retail access on diet in a ‘food desert’: a preliminary report. Urban Stud 39, 2061–2082. |  |
| 29. Gill L & Rudkin S (2014) Deconstructing supermarket intervention effects on fruit and vegetable consumption in areas of limited retail access: evidence from the Seacroft Study. Environ Plann A 46, 649–665. |  |

**Andreyeva et al 2010**

| **Primary studies as referenced in review** | **Primary study also reported by** |
| --- | --- |
| **Food prices** | |
| 18. Huang KS, Lin B. Estimation of Food Demand and Nutrient Elasticities From Household Survey Data. Washington, DC: US Dept of Agriculture; 2000. |  |
| 20. Park JL, Holcomb RB, Raper KC, Capps O Jr. A demand systems analysis of food commodities by US households segmented by income. Am JAgric Econ. 1996;78(2):290–300. |  |
| 34. Bartlett RW. Fluid milk sales related to demand elasticities. JDairy Sci. 1965; 47(12):1314–1321 |  |
| 35. Barnes R, Gillingham R. Demographic effects in demand analysis: estimation of the quadratic expenditure system using microdata. RevEcon Stat. 1984; 66(4):591–601. |  |
| 36. Raper KC, Wanzala MN, Nayga RM Jr. Food expenditures and household demographic composition in the US: a demand systems approach. Appl Econ. 2002;34(8):981–992. |  |

**Backholer et al (2016)**

| **Primary studies as referenced in review** | **Primary study also reported by** |
| --- | --- |
| **Food prices** | |
| 22. Lin BH, Smith TA, Lee JY et al. (2011) Measuring weight outcomes for obesity intervention strategies: the case of a sugar-sweetened beverage tax. Econ Hum Biol9, 329–341. | Thow et al (2014) |
| 28: Sturm R, Powell LM, Chriqui JF et al. (2010) Soda taxes, soft drink consumption, and children’s body mass index. HealthAff (Millwood)29, 1052–1058. | Thow et al (2014)  Olstad et al (2016) |
| 30. Ni Mhurchu C, Eyles H, Schilling C et al. (2013) Food prices and consumer demand: differences across income levels and ethnic groups. PLoS One 8, e75934. |  |
| 31. Finkelstein EA, Zhen C, Nonnemaker Jet al. (2010). Impact of targeted beverage taxes on higher- and lower-income households. Arch Intern Med170, 2028–2034. | Eyles et al (2012)  Thow et al (2014)  McGill et al (2015) |
| 32. Zhen C, Wohlgenant M, Karns Set al. (2011) Habit formation and demand for sugar-sweetened beverages. Am JAgric Econ93, 175–193. | Eyles et al (2012) |
| 33. Briggs AD, Mytton OT, Kehlbacher A et al. (2013) Overall and income specific effect on prevalence of overweight and obesity of 20% sugar sweetened drink tax in UK: econometric and comparative risk assessment modelling study. BMJ347, f6189. |  |
| 34. Briggs AD, Mytton OT, Madden Det al. (2013) The potential impact on obesity of a 10% tax on sugar-sweetened beverages in Ireland, an effect assessment modelling study. BMC Public Health13, 860. |  |
| 36. Zhen C, Finkelstein EA, Nonnemaker Jet al. (2014) Predicting the effects of sugar-sweetened beverage taxes on food and beverage demand in a large demand system. Am JAgric Econ96, 1–25. |  |

**Black et al (2012)**

| **Primary studies as referenced in review** | **Primary study also reported by** |
| --- | --- |
| **Food prices** | |
| 32. Herman DR, Harrison GG, Afifi AA, Jenks E: Effect of a targeted subsidy on intake of fruits and vegetables among low-income women in the special supplemental nutrition program for women, infants, and children. Am JPublic Health2008,98(1):98–105. |  |
| 40. Herman DR, Harrison GG, Jenks E: Choices made by Low-income women provided with an economic supplement for fresh fruit and vegetable purchase. J Am Diet Assoc2006,106(5):740–744. |  |

Cuffey et al (2015)

The references in this study were only visually inspected, because these studies are thematically different from studies in other reviews. The inspection identified no overlapping studies.

**Eyles et al (2012)**

| **Primary studies as referenced in review** | **Primary study also reported by** |
| --- | --- |
| **Food prices** | |
| 29. Allais O, Bertail P, Nichele V (2010) The effects of a fat tax on French households’ purchases: a nutritional approach. Am J Agric Econ 92: 228–244. | McGill et al (2015) |
| 31. Chouinard HH, Davis DE, LaFrance JT, Perloff JM (2007) Fat taxes: big money for small change. Forum Health Econ Policy 10: article 2. | Thow et al (2010) |
| 47. Dong D, Lin B-H (2009) Fruit and vegetable consumption by low-income Americans. Washington (District of Columbia): US Department of Agriculture. | Thow et al (2010) |
| 13. Fantuzzi K (2008) Carbonated soft drink consumption: implications for obesity policy [PhD dissertation]. Storrs (Connecticut): University of Connecticut |  |
| 38. Finkelstein EA, Zhen C, Nonnemaker J, Todd JE (2010) Impact of targeted beverage taxes on higher- and lower-income households. Arch Intern Med 170:2028–2034. | Backholer et al (2016)  McGill et al (2015)  Thow et al (2014) |
| 51. LaCroix A, Muller L, Ruffieux B (2010) To what extent would the poorest consumers nutritionally and socially benefit from a global food tax and subsidy reform? A framed field experiment based on daily food intake. Available: http://ideas.repec.org/p/gbl/wpaper/201004.html. Accessed 25 January 2012. | Thow et al (2014) |
| 40. Nnoaham KE, Sacks G, Rayner M, Mytton O, Gray A (2009) Modelling income group differences in the health and economic impacts of targeted food taxes and subsidies. Int J Epidemiol 38: 1324–1333. | McGill et al (2015)  Thow et al (2010) |
| 48. Nordstrom J, Thunstrom L (2010) Can targeted food taxes and subsidies improve the diet? Distributional effects among income groups. Food Policy 36:259–271. |  |
| 49. Sassi F, Cecchini M, Lauer J, Chisholm D (2009) Improving lifestyles, tackling obesity: the health and economic impact of prevention strategies. OECD Health Working Papers No. 48. doi:10.1787/220087432153 |  |
| 42. Smed S, Jensen J, Denver S (2007) Socio-economic characteristics and the effect of taxation as a healthy policy instrument. Food Policy 32: 624–639. | McGill et al (2015)  Thow et al (2010) |
| 58. Tefft N (2008) The effects of a soft drink tax on household expenditures. Lewiston (Maine): Bates College. Available: http://abacus.bates.edu/,ntefft/research/soft_drink_taxes_ces.pdf. Accessed 28 January 2012. |  |
| 44. Zhen C, Wohlgenant MK, Karns S, Kaufman P (2011) Habit formation and demand for sugar-sweetened beverages. Am J Agric Econ 93: 175–193. | Backholer et al (2016) |

**Hartmann-Boyce (2018)**

| **Primary studies as referenced in review** | **Primary study also reported by** |
| --- | --- |
| **Food prices** | |
| 19. Anderson ES, Winett RA, Bickley PG, Walberg-Rankin J, Moore JF, Leahy M, Harris CE, Gerkin RE. The effects of a multimedia system in supermarkets to alter shoppers’ food purchases: nutritional outcomes and caveats. J Health Psychol 1997; 2:209–23. |  |
| 23. Budd N, Jeffries JK, Jones-Smith J, Kharmats A, McDermott AY, Gittelsohn J. Store-directed price promotions and communications strategies improve healthier food supply and demand: impact results from a randomized controlled, Baltimore City store-intervention trial. Public Health Nutr 2017:1–11. |  |
| 37. Ni Mhurchu C, Blakely T, Jiang Y, Eyles HC, Rodgers A. Effects of price discounts and tailored nutrition education on supermarket purchases: a randomized controlled trial. Am J Clin Nutr 2010;91:736–  47. |  |
| 44. Waterlander WE, Steenhuis IHM, de Boer MR, Schuit AJ, Seidell JC. The effects of a 25% discount on fruits and vegetables: results of a randomized trial in a three-dimensional web-based supermarket. Int JBehav Nutr Phys Act 2012;9:11. |  |
| 45. Waterlander WE, Steenhuis IHM, de Boer MR, Schuit AJ, Seidell JC. Introducing taxes, subsidies or both: the effects of various food pricing strategies in a web-based supermarket randomized trial. Prev Med 2012;54:323–30. |  |
| 47. Waterlander WE, de Boer MR, Schuit AJ, Seidell JC, Steenhuis IHM. Price discounts significantly enhance fruit and vegetable purchases when combined with nutrition education: a randomized controlled supermarket trial. Am J Clin Nutr 2013;97:886–95. |  |
| Labelling | |
| 16. Ducrot P, Julia C, Mejean C, Kesse-Guyot E, Touvier M, Fezeu LK,Hercberg S, Péneau S. Impact of different front-of-pack nutrition labels on consumer purchasing intentions: a randomized controlled trial. AmJ Prev Med 2016;50:627–36. |  |
| 38. Ni Mhurchu CN, Ekaterina V, Yannan J et al. Effects of interpretive nutrition labels on consumer food purchases: the Starlight randomized controlled trial. Am J Clin Nutr 2017;105:695–704. |  |
| Food Retail | |
| 23. Budd N, Jeffries JK, Jones-Smith J, Kharmats A, McDermott AY, Gittelsohn J. Store-directed price promotions and communications strategies improve healthier food supply and demand: impact results from a randomized controlled, Baltimore City store-intervention trial. Public Health Nutr 2017:1–11. |  |
| 33. Lent MR, Vander Veur SS, McCoy TA, Wojtanowski AC, Sandoval B, Sherman S, Komaroff E, Foster GD. A randomized controlled study of a healthy corner store initiative on the purchases of urban, low-income youth. Obesity 2014;22:2494–500. |  |
| 42. Thorndike AN, Bright O-JM, Dimond MA, Fishman R, Levy DE. Choice architecture to promote fruit and vegetable purchases by families participating in the Special Supplemental Program for Women, Infants, and Children (WIC): randomized corner store pilot study. Public Health Nutr 2017;20:1297–305. |  |

**Hendry et al (2015)**

| **Primary studies as referenced in review** | **Primary study also reported by** |
| --- | --- |
| **Food composition** | |
| 25. Angell SY, Cobb LK, Curtis CJ, KontyKJ, Silver LD. Change in trans fatty acid content of fast-food purchases associated with New York City’s restaurant regulation: a pre---post study. Ann Intern Med.2012;157(2):81---86 |  |

**McGill et al (2015)**

| **Primary studies as referenced in review** | **Primary study also reported by** |
| --- | --- |
| **Food prices** | |
| 34. Allais O, Bertail P, Nichèle V. The effects of a fat tax on french households’ purchases: a nutritional approach. Am J Agric Econ. 2010;92:228–45. | Eyles et al (2012) |
| 35. Dallongeville J, Dauchet L, Mouzon O, Réquillart V, Soler L-G. Increasing fruit and vegetable consumption: a cost-effectiveness analysis of public policies. Eur J Pub Health. 2011;21:69–73. | Thow et al (2014) |
| 36. Nederkoorn C, Havermans RC, Giesen JCAH, Jansen A. High tax on high energy dense foods and its effects on the purchase of calories in a supermarket. An experiment Appetite. 2011;56:760–5.’ |  |
| 37. Nnoaham KE, Sacks G, Rayner M, Mytton O, Gray A. Modelling income group differences in the health and economic impacts of targeted food taxes and subsidies. Int J Epidemiol. 2009;38:1324–33 | Eyles et al (2012)  Thow et al (2014) |
| 38. Smed S, Jensen JD, Denver S. Socio-economic characteristics and the effect of taxation as a health policy instrument. Food Policy. 2007;32:624–39. | Eyles et al (2012)  Thow et al (2010) |
| 39. Tiffin R, Salois M. Inequalities in diet and nutrition. Proc Nutr Soc.2012;71:105–11. |  |
| 41. Finkelstein Ea ZC. Impact of targeted beverage taxes on higher- and lower-income households. Arch Intern Med. 2010;170:2028–34. | Backholer et al (2016)  Eyles (2012)  Thow et al (2014) |
| **Food composition** | |
| 49. Millett C, Laverty AA, Stylianou N, Bibbins-Domingo K, Pape UJ. Impacts of a national strategy to reduce population salt intake in England: serial cross-sectional study. PLoS ONE. 7: e29836. |  |

**Nakhimovsky (2016)**

| **Primary studies as referenced in review** | **Primary study also reported by** |
| --- | --- |
| **Food prices** | |
| 35.Colchero MA, Popkin BM ,Rivera JA,Ng. Beverage purchases from stores in Mexico under the excise tax on sugar sweetened beverages: observational study. BMJ. 2016;352:h6704. doi:10.1136/bmj.h6704PMID:26738745. |  |
| 36. Barquera S, Hernandez-Barrera L, Tolentino ML, Espinosa J, Ng SW, Rivera JA, et al. Energy Intake from Beverages Is Increasing among Mexican Adolescents and Adults. Journal of Nutrition. 2008;138(12):2454–61.doi:10.3945/jn.108.092163PMID:19022972 |  |
| 39. Claro RM, Levy RB, Popkin BM, Monteiro CA. Sugar-Sweetened Beverage Taxes in Brazil. American Journal of PublicHealth.2012;102(1):178–83.doi:10.2105/ajph.2011.300313 PMID:22095333 | Thow et al (2014) |
| 40.Basu S, Vellakkal S, Agrawal S, Stuckler D, Popkin B, EbrahimS. Averting Obesity and Type2 Diabetes in India through Sugar-Sweetened Beverage Taxation: An Economic-Epidemiologic Modeling Study. PLoSMedicine. 2014;11(1).e100158210.1371/journal.pmed.1001582.PMID:WOS:000337975600002. |  |
| 44. Paraje G. The Effect of Price and Socio-Economic Level on the Consumption of Sugar-Sweetened Beverages (SSB): The Case of Ecuador.PLoSOne.2016;11(3):e0152260.Epub2016/03/31.doi:10.1371/journal.pone.0152260PMID:27028608. |  |
| 45.Colchero MA, Salgado JC, Unar-Munguı ́a M, Herna ́ndez-A ́vila M, Rivera-Dommarco JA. Price elasticity of the demand for sugar sweetened beverages and soft drinks in Mexico. Economics& HumanBiol-ogy.2015;19:129–37.doi:10.1016/j.ehb.2015.08.007 |  |

#

# Literature list of included systematic reviews

1. Abeykoon AH, Engler-Stringer R, Muhajarine N. Health-related outcomes of new grocery store interventions: a systematic review. *Public health nutrition*. 2017-06-01 2017;20(11):1-13.

2. Andreyeva T, Long MW, Brownell KD. The Impact of Food Prices on Consumption: A Systematic Review of Research on the Price Elasticity of Demand for Food. *American journal of public health*. 2010 2010;100(2):216-222.

3. Backholer K, Sarink D, Beauchamp A, et al. The impact of a tax on sugar-sweetened beverages according to socio-economic position: a systematic review of the evidence. *Public health nutrition*. 2016;19(17):3070-3084. doi:10.1017/s136898001600104x

4. Black AP, Brimblecombe J, Eyles H, Morris P, Vally H, O′Dea K. Food subsidy programs and the health and nutritional status of disadvantaged families in high income countries: a systematic review. *BMC Public Health*. 2012/12/21 2012;12(1):1099. doi:10.1186/1471-2458-12-1099

5. Cuffey J, Beatty TK, Harnack L. The potential impact of Supplemental Nutrition Assistance Program (SNAP) restrictions on expenditures: a systematic review. *Public health nutrition*. 01-Dec 2015;19(17):3216-3231.

6. Eyles H, Ni Mhurchu C, Nghiem N, Blakely T. Food pricing strategies, population diets, and non-communicable disease: a systematic review of simulation studies. *PLoS medicine*. 2012-12-11 2012;9(12):e1001353.

7. Hartmann-Boyce J, Bianchi F, Piernas C, et al. Grocery store interventions to change food purchasing behaviors: a systematic review of randomized controlled trials. *American Journal of Clinical Nutrition*. 06-01 2018;107(6):1004-1016.

8. Hendry VL, Almíron-Roig E, Monsivais P, et al. Impact of Regulatory Interventions to Reduce Intake of Artificial Trans-Fatty Acids: A Systematic Review. *American journal of public health*. 2015-01-20 2015;105(3):e1-e11.

9. McGill R, Anwar E, Orton L, et al. Are interventions to promote healthy eating equally effective for all? Systematic review of socioeconomic inequalities in impact. *BMC Public Health*. 2015/05/02 2015;15(1):457. doi:10.1186/s12889-015-1781-7

10. Nakhimovsky SS, Feigl AB, Avila C, O’Sullivan G, Macgregor-Skinner E, Spranca M. Taxes on Sugar-Sweetened Beverages to Reduce Overweight and Obesity in Middle-Income Countries: A Systematic Review. *PLoS ONE*. 2016 2016;11(9):1-22.

11. Olstad DL, Teychenne M, Minaker LM, et al. Can policy ameliorate socioeconomic inequities in obesity and obesity-related behaviours? A systematic review of the impact of universal policies on adults and children. *Obesity Reviews*. 12 2016;17(12):1198-1217.

12. Olstad DL, Ancilotto R, Teychenne M, et al. Can targeted policies reduce obesity and improve obesity-related behaviours in socioeconomically disadvantaged populations? A systematic review. *Obesity Reviews*. Jul 2017;18(7):791-807.

13. Sarink D, Peeters A, Freak-Poli R, et al. The impact of menu energy labelling across socioeconomic groups: A systematic review. *Appetite*. Apr-01 2016;99:59-75.

14. Schultz DJ, Byker Shanks C, Houghtaling B. The Impact of the 2009 Special Supplemental Nutrition Program for Women, Infants, and Children Food Package Revisions on Participants: A Systematic Review. *Journal of the Academy of Nutrition and Dietetics*. 2015-11-12 2015;115(11):1832-46.

15. Thow AM, Jan S, Leeder S, Swinburn B. The effect of fiscal policy on diet, obesity and chronic disease: a systematic review. *Bulletin of the World Health Organization*. 2010-08-01 2010;88(8):609-14.

16. Thow AM, Downs S, Jan S. A systematic review of the effectiveness of food taxes and subsidies to improve diets: Understanding the recent evidence. *Nutrition reviews*. 2014-08-04 2014;72(9):551-65.
